# Effect of astrocyte GPER on the optic nerve inflammatory response following optic nerve injury in mice

**DOI:** 10.1016/j.heliyon.2024.e29428

**Published:** 2024-04-10

**Authors:** Xuan Wang, Jiaxing Zhou, Yuwen Wang, Xue Li, Qiumei Hu, Linlin Luo, Xuemei Liu, Wei Liu, Jian Ye

**Affiliations:** a Department of Ophthalmology, Daping Hospital, Army Medical Center of PLA, Army Medical University, Chongqing, 400042, China; b Department of Ophthalmology, Xinqiao Hospital, Army Medical University, Xinqiao Road, Shapingba District, Chongqing, 400032, China

**Keywords:** Astrocytes, Glial cells, Central nervous system, Optic nerve injury, Neuropathy, Inflammation

## Abstract

Activated astrocytes are a primary source of inflammatory factors following traumatic optic neuropathy (TON). Accumulation of inflammatory factors in this context leads to increased axonal damage and loss of retinal ganglion cells (RGCs). Therefore, in the present study, we explored the role of the astrocyte G protein-coupled estrogen receptor (GPER) in regulating inflammatory factors following optic nerve crush (ONC), and analyzed its potential regulatory mechanisms. Overall, our results showed that GPER was abundantly expressed in the optic nerve, and co-localized with glial fibrillary acidic proteins (GFAP). Exogenous administration of G-1 led to a significant reduction in astrocyte activation and expression of inflammation-related factors (including IL-1β, TNF-α, NFκB, and *p*-NFκB). Additionally, it dramatically increased the survival of RGCs. In contrast, astrocytes were activated to a greater extent by exogenous G15 administration; however, RGCs survival was significantly reduced. *In vitro*, GPER activation significantly reduced astrocyte activation and the release of inflammation-related factors. In conclusion, activation of astrocyte GPER significantly reduced ONC inflammation levels, and should be explored as a potential target pathway for protecting the optic nerve and RGCs after TON.

## 1 Introduction

Traumatic optic neuropathy (TON) is a pathology resulting from direct or indirect optic nerve crush (ONC), which is associated with a substantial loss of retinal ganglion cells (RGCs) and their axons [1,2]. This loss of RGCs and axonal degeneration can cause partial or permanent visual impairment following injury. Indeed, approximately 50 % of patients with traumatic optic neuropathy experience permanent vision loss, even after clinical treatment, due to the progressive degeneration of RGCs and their axons [3,4].

Astrocytes, which form an intricate network throughout the optic nerve, are the most abundant cells in the central nervous system (CNS) [5]. Following CNS injury, reactive astrocytes become the primary source of inflammatory factors [6,7]. Elevated levels of inflammatory factors are detrimental to the optic nerve in TON, and can lead to further diffuse axonal damage [[8], [9], [10]]. In addition, inflammatory factors activate other glial cells within the optic nerve and mediate glial cell interactions, consequently exacerbating the inflammatory responses, axonal damage, and RGCs loss [11]. Avoiding continuous astrocyte activation and reducing inflammatory factor production can effectively alleviate RGCs loss following ONC [12].

Prior research has shown that 17β-estradiol (E2) can exert potent neuroprotective effects, acting as a significant anti-inflammatory and neuroprotective agent following CNS injury [[13], [14], [15]]. E2, in addition to the classical receptors Eα and Eβ, also exerts potent anti-inflammatory effects while inducing fewer side effects through interaction with atypical G protein-coupled estrogen receptor (GPER), and further interacts with classical receptors in a rapid, non-genomic mode of action [[16], [17], [18]]. Therefore, these are considered essential targets for the treatment of neuropathy. GPER activation protects neurons by regulating astrocyte activation and autophagy, revealing a regulatory role of GPER in astrocytes [19]. However, the specific mechanisms underlying these effects remain unclear. To address this knowledge gap, in the present study, we explored the regulatory role of astrocytic GPER in the inflammatory response following ONC, and revealed its potential molecular mechanisms.

## 2 Materials and methods

### 2.1 Ethical approval

All animal handling procedures followed the policies and guidelines established by the Laboratory Animal Welfare and Ethics Committee of the Third Military Medical University and the Association for Research in Vision and Ophthalmology Animal Statement.

### 2.2 Animal husbandry

Adult C57BL/6J mice (male, aged 6–8 weeks; body weight, 20–24 g) were purchased from the Animal Center of the Army Medical University. All animals were housed in a disease-free facility under a 12/12-h light-dark cycle, with access to food and water provided a*d libitum*.

### 2.3 Optic nerve crush model

Mice were anesthetized via intraperitoneal injection of 1 % sodium pentobarbital, according to the classical ONC model procedure [2,20]. The exposed left optic nerve was crushed 1.5 mm behind the left eyeball for 10 s using ultrafine autoclamps, without damaging the retinal vessels or disturbing the blood supply. The right eye was used as the control. All procedures were performed aseptically, and no incidences of postoperative infection were observed.

### 2.4 Drug treatment

G-1 (GPER agonist; MCE, HY-107216) and G15 (GPER inhibitor; MCE, HY-103449) were solubilized in 10 % corn oil and saline, to produce final injection solutions of 50 μg/kg each. PDTC (NFκB inhibitor; MCE, HY-18738) was administered in a saline configuration with a final injection volume of 50 mg/kg. All injection methods were intraperitoneal. G15 was injected 24 h before ONC to completely inhibit GPER. G-1 was injected 30 min after ONC, simulating drug therapy after ONC. PDTC was injected 30 min before ONC inhibiting NFκB. All drug treatment time selections are based on previous studies [[21], [22], [23]].

### 2.5 Tissue preparation

The optic nerves were dissected and fixed in 4 % paraformaldehyde overnight following heart perfusion. These tissues were subsequently cryopreserved in 30 % sucrose phosphate-buffered saline (PBS) for 1 h, after which tissue embedding was performed, and cry sections of 12 μm thickness were produced. Three sections of optic nerve tissue were randomly selected for immunostaining in most experiments.

Retinal collection was performed as previously described [24]. In brief, the mice were perfused with saline through the apices under anesthesia, and their eyes were removed and placed in 4 % paraformaldehyde (PFA) for 30 min for fixation. The intact retinas were subsequently dissected, cut into four-leaf clover shapes under a microscope, and fixed in ice-cold methanol for 1 h.

### 2.6 Immunofluorescence

Tissue cryosections were incubated with 0.1 % Triton X-100 (Sigma-Aldrich, St Louis, MO, USA) in PBS for 15 min at room temperature, and subsequently incubated with primary antibody at 4 °C overnight. Sections were subsequently incubated with appropriate fluorescent secondary antibodies, including Alexa Fluor594-labeled goat anti-mouse IgG (#8890, 1:500; CST) and Alexa Fluor488 goat anti-rabbit IgG (#4412, 1:500; CST), for 1.5 h at room temperature. Coverslips were blocked after staining of the nuclei with Hoechst stain (ab228551; 1:500, Abcam). Primary astrocytes were fixed with 4 % paraformaldehyde for 15 min at room temperature, and the subsequent staining steps were consistent with those for the tissues. Finally, tissue cryosections and cells were observed under an Olympus confocal microscope.

The primary antibodies used in this study were anti-GFAP (#3670, 1:500, CST) and GPER antibody (GTX107748, 1:200; GeneTex).

### 2.7 Whole-mounted retinal immunofluorescence

The dissected retinas were incubated overnight in goat serum containing 3 % Triton-X100 for containment, after which they were incubated at 4 °C with primary antibodies for 48 h, including anti-TUJ1 (ab18207, 1:500, Abcam) and anti-BRN3A (ab245230, 1:200, Abcam). Subsequently, the retinas were incubated with secondary antibodies at 4 °C for 24 h. Samples were washed with PBS three times for 10 min between each step. Finally, retinas were mounted on slides and observed under an Olympus confocal microscope. Surviving RGCs were manually counted using ImageJ software.

### 2.8 Immunoblotting

We performed Western blot analysis of proteins extracted from freshly dissected optic nerve tissue and cell samples [25]. To collect protein samples, a segment of the optic nerve, approximately 3 mm in length, centered on the extrusion site was removed. Protein samples were run on 10 % SDS-PAGE gels and then transferred to a polyvinylidene fluoride (PVDF) membrane. Following transfer, the membranes were washed and blocked in BSA blocking solution, before incubation with primary antibodies overnight at 4 °C on a shaker. The primary antibodies included GFAP (#3670, 1:1000, CST), NFκB (#8242, 1:1000, CST), *p*-NFκB (#3033, 1:1000, CST), IL-1β (A22257, 1:1000, Abclonal), TNF-α (A20851, 1:500, Abclonal), and GAPDH (AC001, 1:10,000, Abclonal). Membranes were then incubated in appropriate secondary antibodies, including anti-mouse IgG (7076, 1:5000, CST) and anti-rabbit IgG (7074, 1:5000, CST), for 1.5 h at room temperature, and subsequently visualized using an Odyssey Fc Imaging System (Licor, Lincoln, NE, USA). The immunoreactive bands were quantified using ImageJ software to determine the ratio of grayscale values to GAPDH.

### 2.9 Extraction and culture of primary astrocytes

Primary astrocytes were prepared as previously described [26]. One- or two-day-old mice were used. Mice brains were dissected under a microscope, and the cerebral hemispheres were incubated with 0.125 % trypsin-0.05 % ethylenediaminetetraacetic acid for 15 min at 37 °C. Digestion was terminated by addition of Dulbecco's modified Eagle's medium containing 10 % fetal bovine serum, after which brain tissues were dissociated and dispersed into individual cells, and inoculated at a density of 1 × 10^4^/cm^2^ in polylysine pre-coated 75 cm^2^ Petri dishes. The medium was then replaced with fresh, complete medium, and the remaining suspension cells were removed after 24 h. Cells were cultured in an incubator at 37 °C, 95 % air, and 5 % CO2 for approximately 7 days to achieve 90 % confluency of the cell growth area. Petri dishes were rotated at 260 rpm (24 h, 37 °C) to collect the purified astrocytes. The cells exhibited >98 % GFAP-positive staining.

### 2.10 Pharmacologic treatment of primary astrocytes

Primary astrocytes were cultured and passaged until the third generation, after which they were divided into different treatment groups. The control and DMSO groups were left untreated and treated with 0.1 % DMSO for 2.5 h, respectively. Cells in the GLU group were exposed to 300 μM glutamate for 1 h. Primary astrocytes in the GLU + G-1 group were pretreated with 10 nM G-1 for 30 min. Subsequently, glutamate-exposed cells were continuously treated with G-1 for 1 h. Cells in the GLU + G15 group were pretreated with G15 for 90 min, after which glutamate treatment was continued for 1 h. The GLU + G-1 + G15 group was administered as follows: G15 was added, after which the subsequent treatment was similar to that in the G-1 group. PDTC was administered 30 min before glutamate treatment. G-1 and G15 were dissolved in DMSO to 100 mM and finally diluted in the culture medium (the final DMSO concentration was ≤0.1 %). PDTC was dissolved in sterile water to a concentration of 100 μM immediately before use.

### 2.11 Enzyme-linked immunosorbent assay (ELISA)

The supernatant of primary cultured was collected, and the levels if inflammatory factors were assessed using a commercial IL-1β ELISA kit (R&D Systems, MLB00C) and TNF-α ELISA kit (R&D Systems, MTA00B), according to the manufacturer's protocol.

### 2.12 Statistical analysis

Statistical analyses were performed using GraphPad Prism 7.0 software (GraphPad, La Jolla, CA, USA), with results reported as the mean ± SEM. Statistical comparisons were performed using two-way analysis of variance (ANOVA), followed by Tukey's post hoc test to compare between multiple groups. Statistical significance was set at P < 0.05. All groups in all experiments contained at least three biological replicates (n ≥ 3). All experiments were conducted independently in triplicate or more.

## 3 Results

### 3.1 GPER is negatively correlated with astrocyte activation

**Fig. 1:**
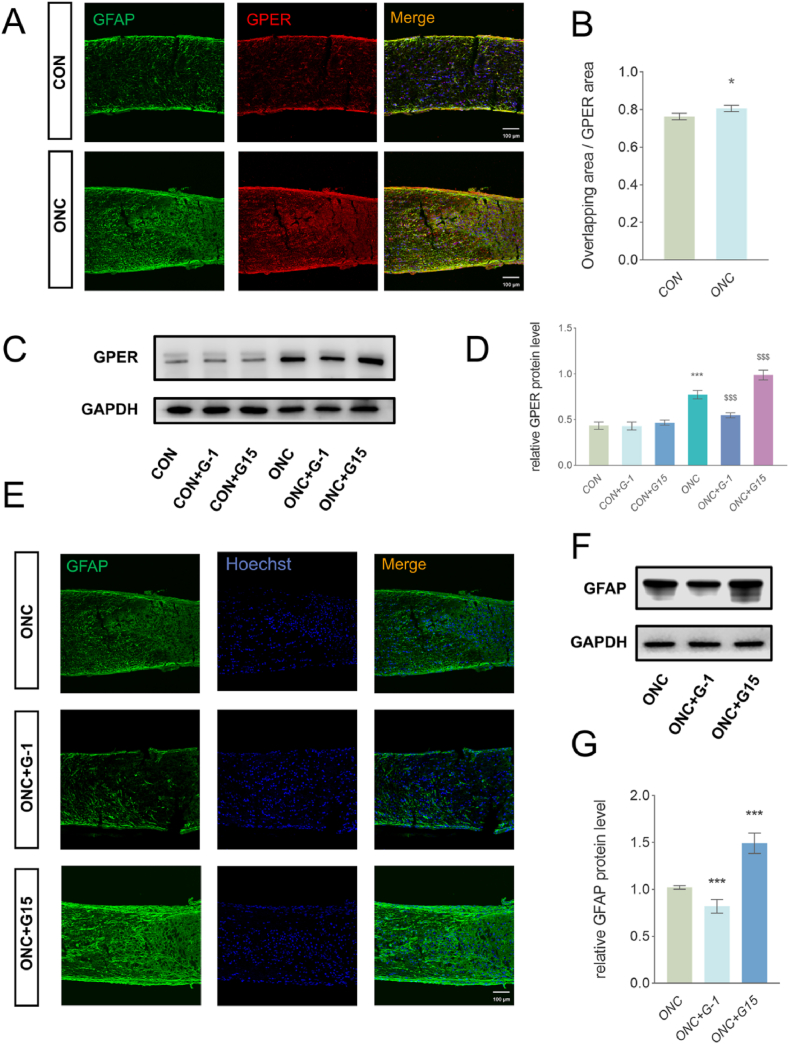
Effect of GPER G-1 activation or G15 inhibition on astrocyte activation. (A) Representative images of GPER (red) and GFAP (green) with Hoechst (blue) immunofluorescence (IF) staining of the optic nerve in the control group and ONC group. Scale bar = 100 μm. (B) Co-localization analysis of GPER and GFAP. *P < 0.05. (C, D) Western blotting (WB) and quantitative analyses revealed that G-1 and G15 treatment significantly decreased and elevated GPER expressing around the ONC site 7 dpi. While no significant difference was observed in groups without ONC. GAPDH was used as a loading control. The data shows the mean value of the gray value ratio from three independent experiments (mean ± SEM, n = 3 mice per group). ***P < 0.001 vs. the CON group. ^$$$^ P < 0.001 vs. the ONC group. (E) Representative images of GFAP (green) with Hoechst (blue) IF staining around the ONC site after ONC 7 days after G-1 or G15 use. Scale bar = 100 μm. (F) WB showing the GFAP expression around the ONC site 7 dpi after GPER was activated or inhibited. GAPDH was used as a loading control. (G) In a quantitative analysis of GFAP protein expression, G-1 significantly reduced GFAP expression and G15 significantly elevated GFAP expression. The data shows the mean value of the gray value ratio from three independent experiments (mean ± SEM, n = 3–4 mice per group). ***P < 0.001 vs. the ONC group.

We investigated the potential role of GPER in ONC by localizing its distribution in the optic nerve. The expression pattern of GPER was analyzed in ON sections by immunofluorescence (IF) (Fig. 1A and B). GPER showed high co-localization with the astrocyte marker GFAP. The ratio of GPER area colocalized with the GFAP to total GPER area was 0.7640 in the CON group, and this ratio increased significantly to 0,8063 after ONC. The results suggest that there is substantial GPER expression in astrocytes, consistent with a previous report [27]. After G-1 and G15 treatment, optic nerve GPER significantly decreased and elevated after ONC 7 days (Fig. 1C and D). And retina GPER experienced the same state (Supplementary Fig. 1). While there was no significant difference in the CON group. We also examined optic nerve GFAP expression and the number of RGCs after injection of G-1 and G15 in the CON group, no significant difference was observed, either (Supplementary Fig. 2).

GPER is abundant in the optic nerve and co-localizes with astrocytes, suggesting that GPER may be involved in regulating astrocyte activation in ONC. Our study revealed that GPER activation by G-1 and inhibition by G15 significantly decreased and increased GFAP expression in astrocytes, respectively (Fig. 1E, F and G). These results suggested that GPER plays an important role in regulating post-injury optic nerve. In addition, GPER activation attenuates astrocyte activation.

### 3.2 GPER is involved in regulating the production of inflammatory factors after ONC

**Fig. 2:**
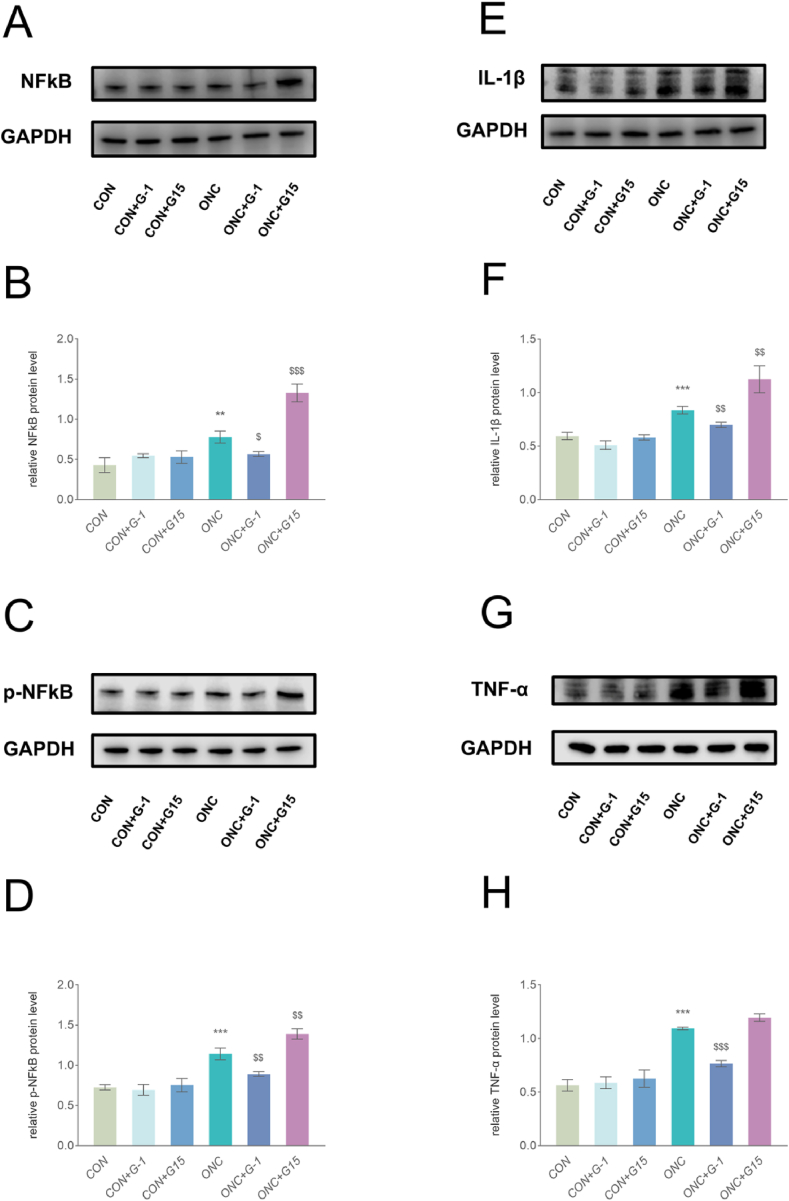
Effects of different GPER activation states on the levels of inflammation-related factors. (A–D) WB and quantitative analyses revealed that G-1 and G15 treatment significantly decreased and elevated NFkB and *p*-NFkB expression at day 7 of ONC, respectively. The results represent three independent experiments and are shown as mean ± SEM (n = 3–4 mice per group). (E–H) WB and quantitative analyses revealed that G-1 and G15 treatment significantly decreased and elevated IL-1βand TNF-α expression at day 7 of ONC, respectively. The results represent three independent experiments and are shown as mean ± SEM (n = 3–4 mice per group). **P < 0.01 and ***P < 0.001 vs. the CON group. ^$^ P < 0.05, ^$$^ P < 0.01 and ^$$$^ P < 0.001 vs. the ONC group.

The NFκB factor has been shown to regulate astrocyte activation in multiple eye diseases [[28], [29], [30]]. We examined the changes in the expression of inflammatory pathway-related factors (NFκB and *p*-NFκB) by WB (Fig. 2A, B, C and D), to verify whether GPER also regulates astrocyte activation through NFκB. GPER activation by G-1 and its inhibition by G15 induced a significant reduction and increase reduction of the expression levels of NFκB and *p*-NFκB, respectively. This suggests that GPER activation decreases NFκB expression.

Reactive astrocytes after ONC are major sources of inflammatory factors, while astrocyte NFκB activation stimulates the production of inflammatory factors, including the representative inflammatory factors TNF-α and IL-1β [31]. In contrast, uncontrolled inflammatory cascade responses aggravate this response [11]. GPER activation modulates NFκB expression and inhibits astrocyte activation; therefore, we examined the effect of GPER on inflammatory factors in the optic nerve after ONC. We further examined the changes in IL-1β and TNF-α levels (Fig. 2E, F, G and H). GPER activation significantly decreased IL-1β and TNF-α expression levels. In contrast, GPER inhibition significantly increased IL-1β expression. However, no significant difference in TNF-α expression was observed following optic nerve entrapment alone (P = 0.1309). These results suggested that GPER activation reduced the expression of inflammatory factors.

### 3.3 GPER promotes RGCs survival after injury

**Fig. 3:**
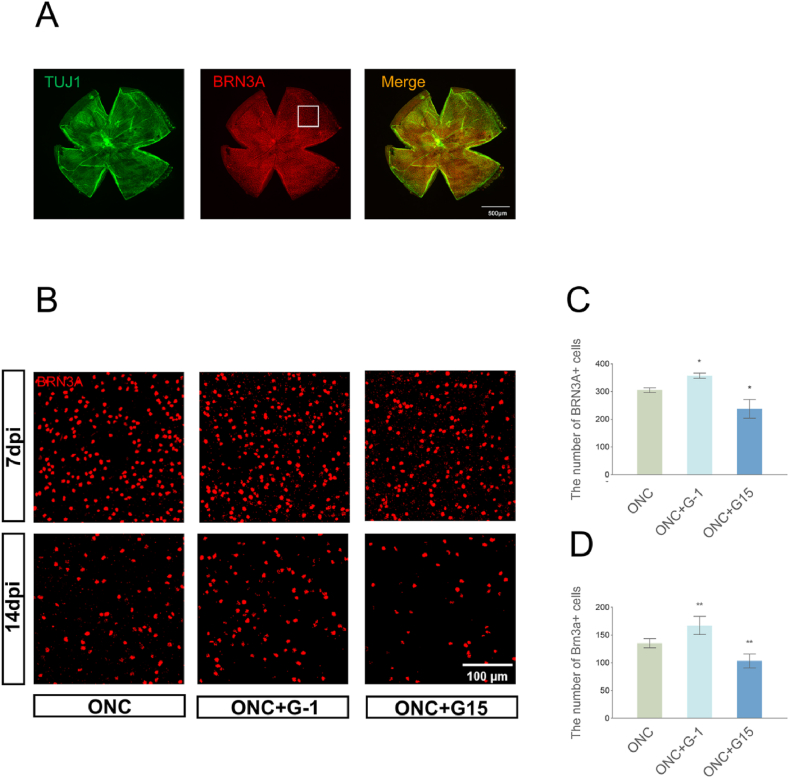
Effect of GPER activation or inhibition on the number of surviving retinal RGCs after ONC. (A) Representative images of IF staining for the retinal neuronal marker TUJ1 (green), and the RGCs marker BRN3A (red) in the sham-operated group. Four 300-μm-sized square areas in the peripheral part of each sector of the retina were selected for cell counting, and the average value was considered to represent the number of RGCs surviving in that retina for statistical analysis. Scale bar = 500 μm. (B) Representative IF maps of BRN3A in the periphery. Scale bar = 100 μm. (C–D) Counts of BRN3A-positive cells in the ONC retina on days 7 (Fig. 3C) and day 14 (Fig. 3D). G-1 treatment significantly elevated the number of RGCs at both timepoints, while G15 treatment significantly decreased the number of RGCs. The average number of BRN3A-positive cells of 4 fields was used as the data and are shown as mean ± SEM (n = 4–5 mice per group). *P < 0.05 and **P < 0.01 vs. the ONC group.

One prior study has indicated that reducing the reactivation of astrocytes and the levels of inflammation following ONC may reduce the loss of RGCs [10]. Therefore, we examined the effect of GPER on RGCs survival after ONC. We assessed the co-staining of the neuronal cell marker TUJ1 with the RGCs nuclei marker BRN3A (Fig. 3A) to count the number of RGCs in 300 × 300 (μm) square areas selected from each sector of the four-leaf clover-shaped retina at the positions shown in Fig. 3A, with four areas per retina. We further compared the difference in the number of surviving RGCs between the G-1 and G15 groups 7 and 14 days after ONC with the number of RGCs after ONC alone (Fig. 3B, C and D). The survival rate of patients with RGCs significantly increased and decreased after G-1 and G15 injections, respectively, 7 and 14 days after ONC. In addition, GPER activation exhibits protective properties and significantly reduces the loss of RGCs after ONC. Meanwhile the TUJ-1 results consistent with BRN3A quantifications (Supplementary Fig. 3).

### 3.4 GPER activation reduced glutamate-induced primary astrocyte activation *ex vivo*

GPER is expressed in astrocytes of the optic nerve, microglia, and neurons [32,33]. Therefore, we performed *in vitro* experiments to verify the effects of GPER on astrocytes. After ONC, astrocytes were activated using glutamate to mimic astrocyte activation. Subsequently, G-1 or G15 was added to the culture medium to simulate different GPER activation states, and to observe the regulatory effect of GPER on astrocyte activation.

**Fig. 4:**
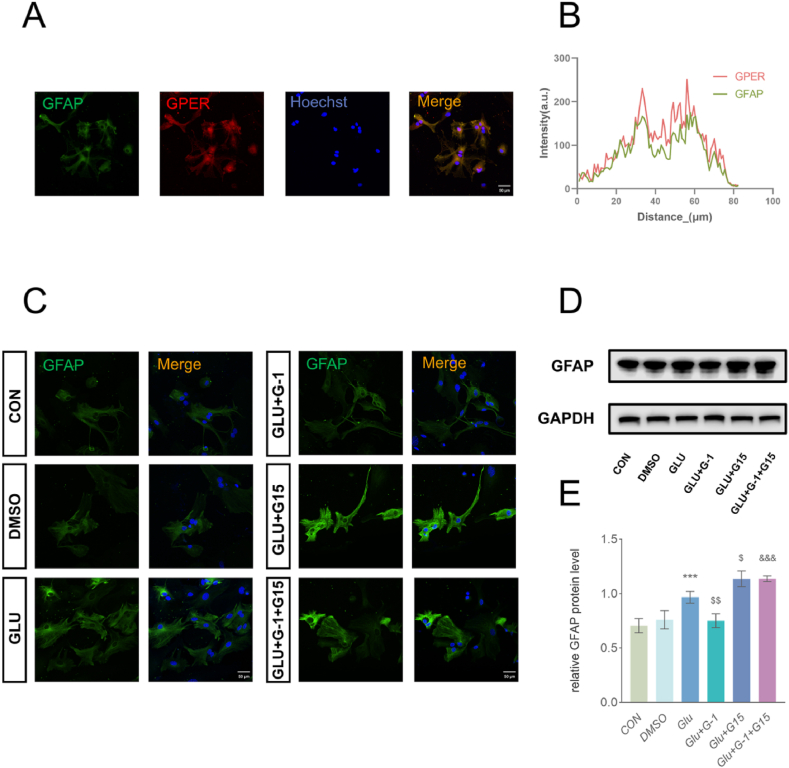
GPER regulates the activation of primary astrocytes. (A) Representative immunofluorescent images of primary astrocytes stained with GPER (red) and GFAP (green) with Hoechst (blue). Scale bar = 50 μm. (B) Co-localization analysis of GPER and GFAP. Pearson's Coefficient: r = 0.847. (C) Representative IF staining images of glutamate-activated primary astrocytes with GFAP with Hoechst in different states of GPER. Scale bar = 50 μm. (D–E) WB and quantitative analyses revealed that dimethyl sulfoxide (DMSO) had no significant effect on GFAP expression in primary astrocytes, glutamate significantly elevated GFAP expression, and G-1 use significantly decreased GFAP expression in astrocytes following glutamate activation, and vice versa for G15. Additionally, G15, combined with G-1, reversed the effect of G-1. Data are presented as the mean values of three independent experiments (mean ± SEM, n = 3 wells per group). ***P < 0.001 vs. the DMSO group; ^$^ P < 0.05 and ^$$^ P < 0.01 vs. the GLU group; ^&&&^ P < 0.001 vs. the GLU + G-1.

IF analyses revealed that GPER co-localized with GFAP (Fig. 4A and B). Subsequently, we examined the effects of different states of GPER activation on astrocyte activation using IF and WB (Fig. 4C, D and E). Overall, our results showed that treatment with DMSO alone did not activate astrocytes, whereas glutamate treatment activated astrocytes, resulting in a significantly higher GFAP protein expression and fluorescence intensity. G-1 and G15 pretreatments significantly inhibited and elevated astrocyte activation, respectively. Consequently, G15 reversed the G-1 attenuation of astrocyte activation, which was significantly elevated in the G-1 + G15 group compared to that in the G-1 group.

### 3.5 GPER activation reduced the levels of inflammatory factors released following primary astrocyte activation

**Fig. 5:**
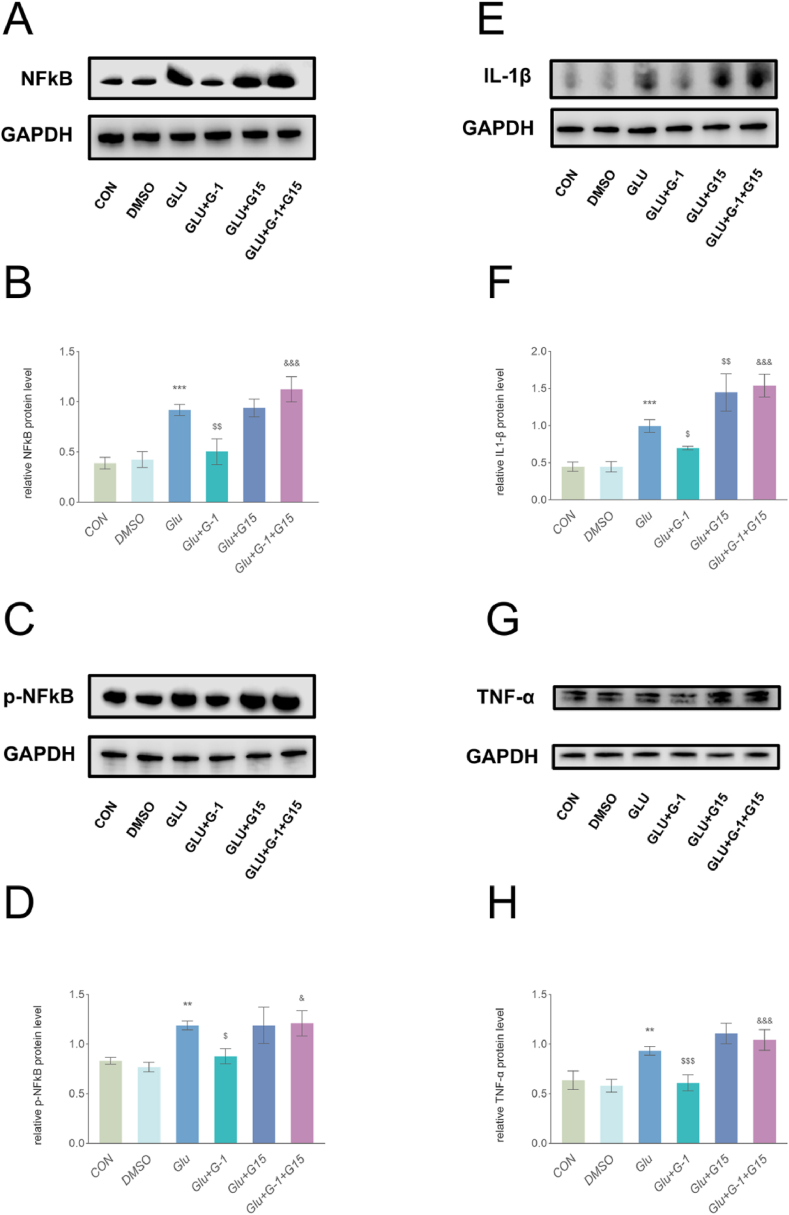
GPER regulates the release of inflammation-related factors following primary astrocyte activation. (A–D) WB and quantitative analyses revealed that G-1 significantly decreased NFkB and *p*-NFkB expression in primary astrocytes after glutamate activation, G15 alone did not cause significant changes, and the combination of G15 and G-1 significantly attenuated the effect of G-1. Data are presented as the mean values of three independent experiments (mean ± SEM, n = 3 wells per group). (E–H) WB and quantitative analyses revealed that G-1 significantly decreased IL-1β and TNF-α expression in primary astrocytes following glutamate activation, G15 alone significantly elevated IL-1β expression, and the combination of G15 and G-1 also significantly attenuated the effect of G-1. Data are presented as the mean values of three independent experiments (mean ± SEM, n = 3 wells per group). **P < 0.01 and ***P < 0.001 vs. the DMSO group; ^$^ P < 0.05, ^$$^ P < 0.01 and ^$$$^ P < 0.001 vs. the GLU group; ^&^ P < 0.05 and ^&&&^ P＜0.001 vs. the GLU + G-1 group.

We further examined the expression of inflammatory pathway-related factors (NFκB and *p*-NFκB) and inflammatory factors (IL-1β and TNF-α). WB revealed that NFκB expression significantly increased after glutamate activation of astrocytes, while treatment with DMSO alone did not cause changes in NFκB expression (Fig. 5A, B, C and D). Conversely, treatment with G-1 significantly inhibited the NFκB expression of primary astrocytes following glutamate activation, while treatment with G15 reversed the G-1 reduction effect of NFκB and *p*-NFκB.

WB results further showed that G-1 also significantly reduced IL-1β and TNF-α release from primary astrocytes. G-1+G15 treatment reversed the G-1 attenuation of inflammatory factor release compared with G-1 treatment alone (Fig. 5E, F, G and H). Thus, these results suggest that GPER activation controls the release of inflammatory factors, whereas inhibition of GPER activation reverses this effect.

### 3.6 GPER regulates the activation of primary astrocytes and the inflammatory factors via NFκB

**Fig. 6:**
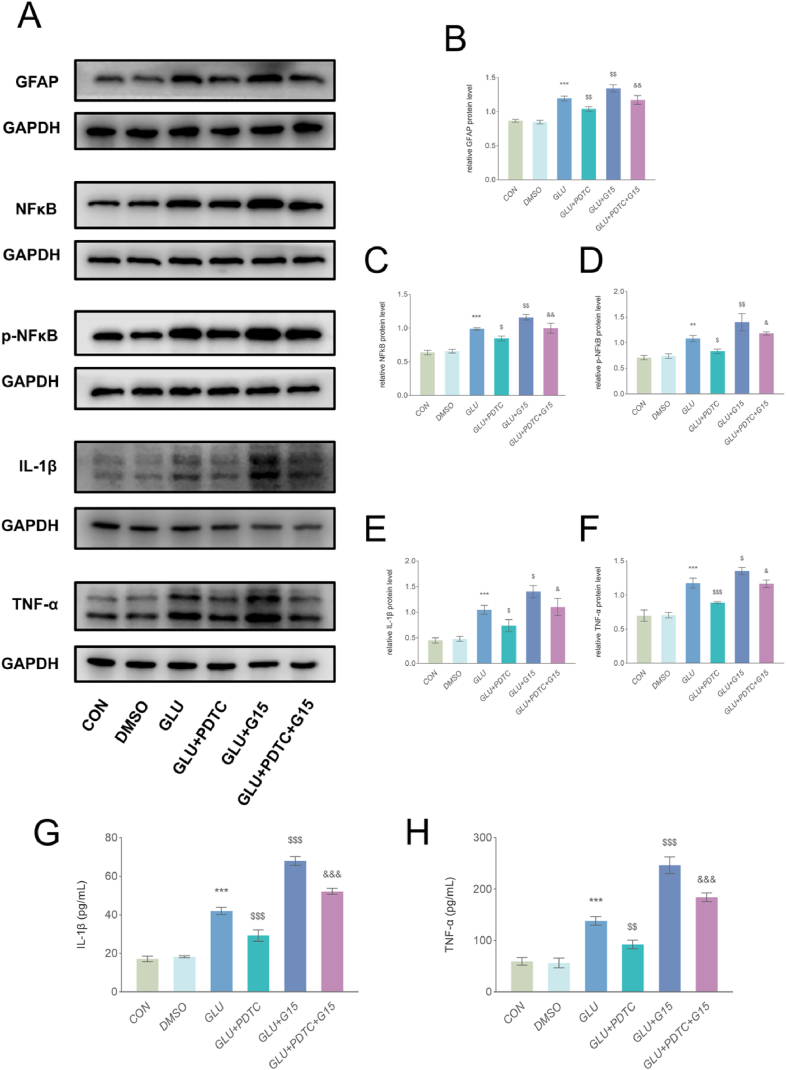
GPER regulates the activation of primary astrocytes via NFκB. (A–F) WB and quantitative analyses revealed that PDTC significantly decreased GFAP, NFκB, *p*-NFκB, IL-1β, and TNF-α expression in primary astrocytes after glutamate activation, and vice versa for G15. The combination of G15 and PDTC significantly attenuated the effect of G15. Data are presented as the mean values of three independent experiments (mean ± SEM, n = 3 wells per group). (G–H) The ELISA results showed that the levels of IL-1β and TNF-α were significantly increased by G15 treatment, while PDTC would block the promoting effect of G15. Data are presented as the mean values of three independent experiments (mean ± SEM, n = 3 wells per group). **P < 0.01 and ***P < 0.001 vs. the DMSO group; ^$^ P < 0.05, ^$$^ P < 0.01 and ^$$$^ P < 0.001 vs. the GLU group; ^&^ P < 0.05 and ^&&&^ P ＜0.001 vs. the GLU + G15 group.

Subsequently, we used the specific NFκB inhibitor PDTC to further explore whether GPER regulates the activation of primary astrocytes and the release of inflammatory factors via the NFκB pathway (Fig. 6A, B, C, D, E, F, G and H). WB further showed that PDTC treatment significantly reduced the expression of GFAP in glutamate-activated primary astrocytes, indicating that PDTC inhibits astrocyte activation. However, compared to the Glu + G15 group, the results from the Glu + G15 + PDTC group indicated that PDTC blocked the activation effect of G15 on astrocytes.

The WB results further showed that the expression of NFκB, *p*-NFκB and its downstream inflammatory factors, IL-1β and TNF-α were significantly decreased in the Glu + PDTC group compared with the Glu group. The promoting effect of G15 on NFκB, *p*-NFκB, IL-1β, and TNF-α was blocked by PDTC. The ELISA results were consistent with WB results, showing that the levels of the inflammatory factors IL-1β and TNF-α were significantly increased by G15 treatment, while PDTC would block the promoting effect of G15 on IL-1β and TNF-α.

### 3.7 GPER regulates the activation of astrocytes via NFκB *in vivo*

**Fig. 7:**
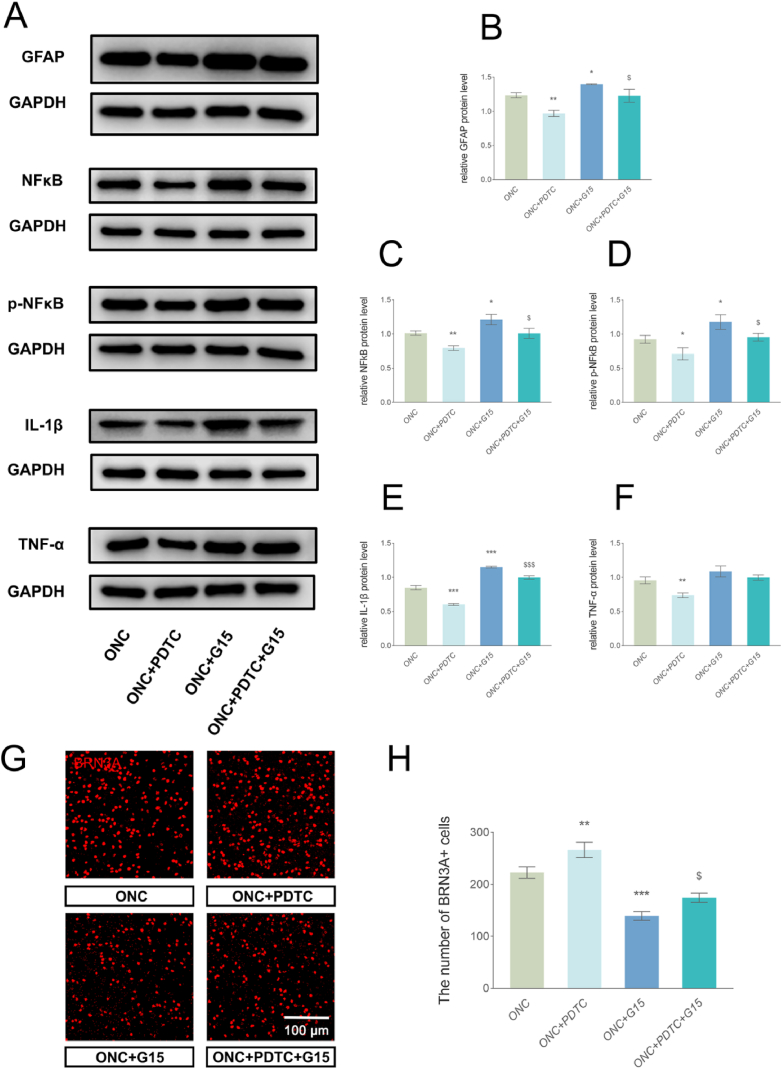
GPER regulates the activation of astrocytes via NFκB. (A–F) WB and quantitative analyses revealed that PDTC significantly decreased GFAP, NFκB, *p*-NFκB, IL-1β, and TNF-α expression after ONC, and G15 significantly increased GFAP, NFκB, *p*-NFκB and IL-1βexpression. The combination of G15 and PDTC significantly attenuated the effect of G15. Data are presented as the mean values of three independent experiments (mean ± SEM, n = 3 mice per group). (G) Representative IF maps of BRN3A in the periphery after ONC. Scale bar = 100 μm. (H) Counts of BRN3A-positive cells in the ONC retina on days 7. PDTC treatment significantly elevated the number of RGCs, while G15 treatment significantly decreased the number of RGCs. The deleterious effect of G15 on the survival of RGCs after ONC was inhibited by PDTC. Data are presented as the mean values of three independent experiments (mean ± SEM, n = 3 mice per group). *P < 0.05, **P < 0.01 and ***P < 0.001 vs. the ONC group; ^$^ P < 0.05 and ^$$$^ P < 0.001 vs. the ONC + G15 group.

The role of GPER identified in our *in vitro* experiments was further validated in mice. WB of optic nerve samples showed that the intraperitoneal injection of the NFκB inhibitor PDTC reduced the expression of GFAP in optic nerve astrocytes after ONC. GFAP expression was significantly reduced in the optic nerve of the G15 + PDTC group compared to that in the G15 group. Meanwhile, the expression of NFκB, *p*-NFκB and the inflammatory factors IL-1β in the optic nerve of the G15 + PDTC group were also significantly decreased compared with the G15 group. This indicates that the effects of G15 in promoting astrocyte activation and inflammatory factor release were blocked by PDTC (Fig. 7A, B, C, D, E and F).

At day 7 post-ONC, the number of retinal RGCs in the G15 + PDTC group was also significantly higher than that in the G15 group, indicating that the deleterious effect of G15 on the survival of RGCs after injury was inhibited by PDTC (Fig. 7G and H). The above results suggest further evidence that the inhibition of GPER following optic nerve injury exerts adverse effects on the optic nerve and retinal RGCs through activation of the NFκB pathway.

## 4 Discussion

The incidence of TON has shown an increasing trend in recent years [34], meaning that the identification of new mechanisms to protect the optic nerve is of paramount importance. Elevated levels of inflammatory factors after ONC is an important contributor to axonal destruction and RGCs death [35]; therefore, modulating inflammation levels may protect the optic nerve after injury.

GPER is a G protein transmembrane receptor abundantly distributed in CNS astrocytes, microglia, and neuronal cells [36,37]. Compared to other receptors in this class, GPER is a fast-acting atypical estrogen receptor. Additionally, GPER exerts neuroprotective effects without the carcinogenic risks associated with traditional estrogen receptors [[38], [39], [40]]. GPER mediates the acute neuroprotective effects of estrogen and has broad clinical applications [41]. GPER also regulates neuronal functions such as neurotransmitter release and hippocampal synaptic plasticity [27,42]. The acute application of G-1 following ischemic injury has also been shown to reduce neuronal damage in adult rats and mice [27,43,44]. GPER activation attenuates CNS damage and exerts a neuroprotective effect. However, its role in traumatic optic neuropathy has not yet been explored, and its mechanism of action remains unclear. Therefore, we investigated the potential neuroprotective effects and mechanisms of action of astrocytic GPER following ONC.

Astrocytes are the most abundant type of glial cell in the CNS [45,46]. These cells undergo a complex series of changes to develop into reactive astrocytes following CNS injury-induced damage [47]. Reactive astrocytes are categorized into harmful type A1 astrocytes and beneficial type A2 astrocytes [48,49]. The elevated levels of pro-inflammatory factors secreted by A1 astrocytes contribute to sustained microglial activation, amplifying the primary ONC effects, and leading to limited nerve damage repair [50]. GPER is abundant in the CNS, and its activation significantly reduces astrocyte activation in models of traumatic brain injury, cerebral ischemia, and other neuroprotective receptors [19,51]. Our study revealed that GPER was abundant in the optic nerve, and colocalized with GFAP, which is highly expressed in astrocytes, indicating its involvement in ONC regulation. In vivo, astrocytes have the possibility of regulating microglia, but a study found that microglia depletion with PLX after ONC does not affect RGCs survival [52], so more studies are needed to illustrate the role of microglia after optic nerve injury. Overall, the results of our study demonstrated that GPER activation significantly reduces astrocyte activation following ONC, as observed in CNS injury.

The NFκB signaling pathway regulates astrocyte activation in a variety of different brain injuries [53,54]. Furthermore, inhibition of the A1 phenotype and activation of the NFκB pathway during CNS injury has been shown to inhibit inflammatory factor release and neuronal apoptosis [[55], [56], [57]]. Similarly, in ocular diseases, astrocyte activation and changes in gene expression in various optic nerves and retinal degenerative diseases are associated with NFκB signaling pathway activation [58,59]. In our study, we observed that the expression levels of NFκB and *p*-NFκB were significantly reduced by GPER activation, indicating that GPER may reduce astrocyte activation by inhibiting the NFκB signaling pathway.

Upon activation, A1-type astrocytes lose their function, release large amounts of inflammatory factors, and induce neuronal death in brain injury disease models [60,61]. Activated type A1 astrocytes drive an increased secretion of pro-inflammatory factors, high inflammation levels, and increased optic nerve damage following ONC [62]. Additionally, the combined effect of IL-1 and TNF induces the production of type A1 astrocytes [49,56]. TNF-α and IL-1β are representative inflammatory factors secreted in large quantities by type A1 astrocytes following ONC [31]. Our study revealed that GPER activation significantly alleviated optic nerve astrocyte activation in the context of ONC, while TNF-α and IL-1β exhibited anti-inflammatory effects by reducing their expression on the optic nerve, suggesting that GPER activation attenuated the inflammatory response following optic ONC.

Many RGCs are lost following ONC, resulting in blindness [63]. Reactive astrocyte accumulation may explain the underlying apoptotic processes in RGCs following TON [64]. Our results were similar to those of a previous study that revealed that astrocytes were activated, while their marker, GFAP, was substantially expressed on day 3 (Supplementary Fig. 1), with many apoptotic RGCs observed by day 7 in the ONC model (Supplementary Fig. 2) [5,65]. We further examined the changes in the number of surviving RGCs after ONC in response to the neuroprotective effects of GPER. The survival of RGCs significantly increased at 7 and 14 days after GPER activation, indicating that GPER activation is neuroprotective and prevents further damage to RGCs. This result agrees with that of a previous report, which found that GPER activation significantly reduced neuronal damage after traumatic brain injury [66]. Furthermore, GPER inhibition using G15 significantly reduced RGCs survival, suggesting that GPER indirectly regulates RGCs following ONC.

Despite these results, it should be noted that GPER expression is not specific to the optic nerve [36,37], and GPER activation may therefore exert indirect effects on neurons and other glial cells. Our *in vitro* experiments to validate the effects of GPER on primary astrocytes showed that GPER activation significantly attenuated the glutamate-mediated activation of primary astrocytes. IL-1β, TNF-α, and NFκB expression levels were all significantly reduced, consistent with the results of *in vivo* experiments, which corroborated the hypothesis that GPER activation in astrocytes directly attenuates astrocyte activation and the release of inflammatory factors. Our results further indicated that this effect may involve modulation of the NFκB pathway.

Subsequently, we applied the specific NFκB inhibitor PDTC to further explore the regulatory mechanism of GPER. The results of our *in vivo* experiments were consistent with the *in vitro* results, in that the increased levels of reactive astrocytes, activation of NFκB pathway, and increased effect of inflammatory factors caused by GPER inhibition were blocked by PDTC. Meanwhile, the combination of PDTC and G15 significantly increased the survival of RGCs following ONC compared with G15 treatment alone. This further proves that GPER regulates the activation of astrocytes and the release of inflammatory factors through modulation of the NFκB pathway, thus affecting the outcomes of ONC.

## 5 Conclusion

In conclusion, the results of the present study suggest that GPER activation on astrocytes in ONC exerts a protective effect on RGCs, possibly through the modulation of the NFκB pathway. These results promote GPER as a potential target for ONC treatment. However, complex interactions exist between cells in ONC. Furthermore, our study has some limitations. For example, the specific mechanism of GPER regulation of astrocyte activation and the specific survival promotion mechanism of RGCs after the reduction of inflammatory factors have not yet been clearly defined, highlighting the need for further investigation.

## Ethical statement

This study was performed with approval from The Laboratory Animal WeIfare and Ethics Committee of Third Military Medical University (AMUWEC20210908).

## Funding

This research was funded by the National Natural Science Foundation of China (No. 82171054).

## Data availability

The data associated with this study has not been deposited into a publicly available repository due to the requirements by project funders. However, the data will be made available on request.

## CRediT authorship contribution statement

**Xuan Wang:** Writing – review & editing, Writing – original draft, Visualization, Validation, Software, Methodology, Investigation, Formal analysis, Data curation, Conceptualization. **Jiaxing Zhou:** Writing – review & editing, Visualization, Validation, Software, Methodology, Investigation, Formal analysis, Data curation. **Yuwen Wang:** Software, Investigation, Data curation. **Xue Li:** Investigation, Data curation. **Qiumei Hu:** Investigation, Data curation. **Linlin Luo:** Investigation, Data curation. **Xuemei Liu:** Investigation, Data curation. **Wei Liu:** Supervision, Resources, Project administration, Funding acquisition, Conceptualization. **Jian Ye:** Supervision, Resources, Project administration, Funding acquisition, Conceptualization.

## Declaration of competing interest

The authors declare that they have no known competing financial interests or personal relationships that could have appeared to influence the work reported in this paper.

## Abbreviations

traumatic optic neuropathy: TON
retinal ganglion cells: RGCs
G protein-coupled estrogen receptor: GPER1
glial fibrillary acidic protein: GFAP
optic nerve crush: ONC
Dimethyl sulfoxide: DMSO
