# Supplementary material for: Effect of astrocyte GPER on the optic nerve inflammatory response following optic nerve injury in mice
Source: Heliyon. 2024 Apr 10;10(8):e29428. doi: 10.1016/j.heliyon.2024.e29428 (PMC11024623; doi:10.1016/j.heliyon.2024.e29428)
Supplement: Multimedia component 1 [file mmc1.docx]

Supplementary materials

SFig 1 Detection of GFAP and GPER expression in retina by IF. A) Representative images of GFAP (green) and GPER (red) IF staining in retina after ONC.Scale bar=100 µm. B) The quantitative analysis shows that Average fluorescence intensity of GPER increased and decreased significantly by G-1 and G15 . *P < 0.05 vs. the CON group. Data are presented as the mean values of three independent experiments (mean ± SEM, n = 4 mice per group). ^$^P < 0.05 and ^$$$^P< 0.001 vs. the ONC group.


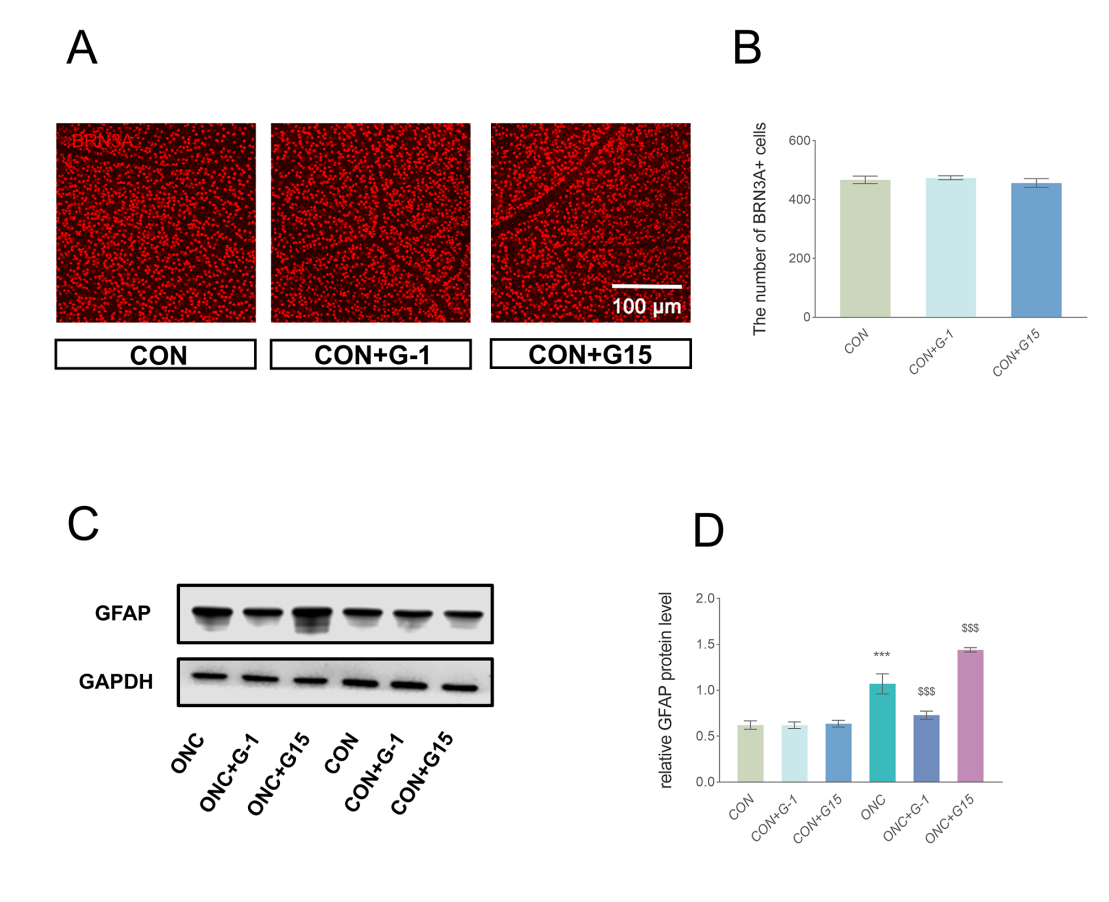


SFig 2 Detection of GFAP and BRN3A expression in the CON group. A) Representative images of BRN3A (red) IF staining in retina .Scale bar=100 µm. B) The quantitative analysis shows no difference. C - D )WB and quantitative analyses revealed that no difference between the CON group. Data are presented as the mean values of three independent experiments (mean ± SEM, n = 3 mice per group). ***P < 0.001 vs. the CON group. $$$ P < 0.001 vs. the ONC group.


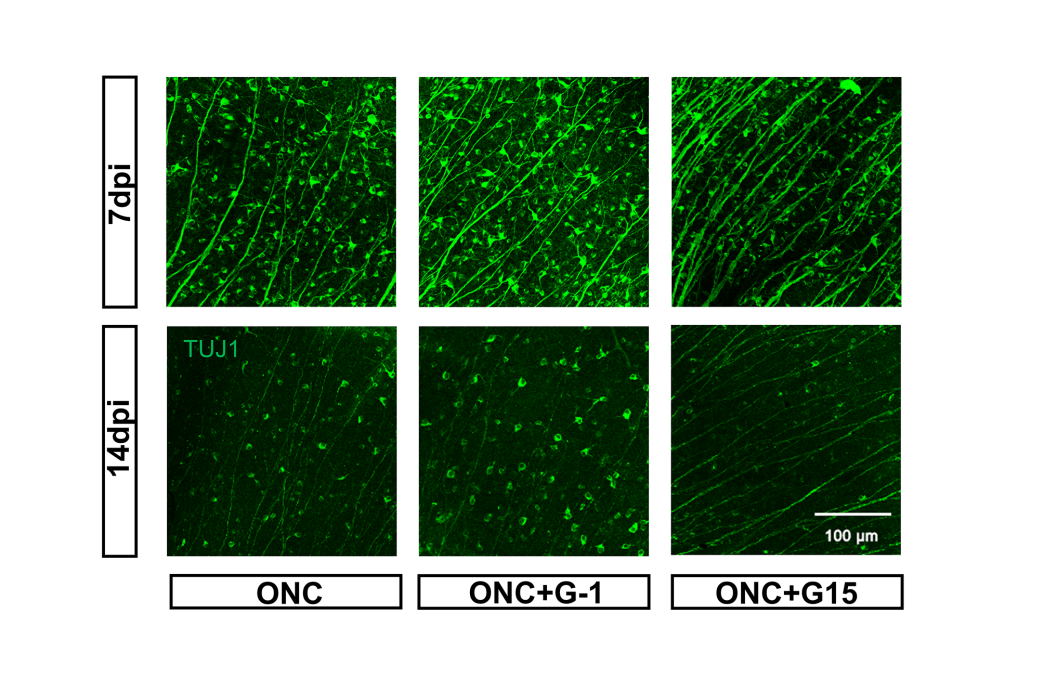


SFig 3 Representative images of TUJ-1 (green) IF staining in retina .Scale bar=100 µm.


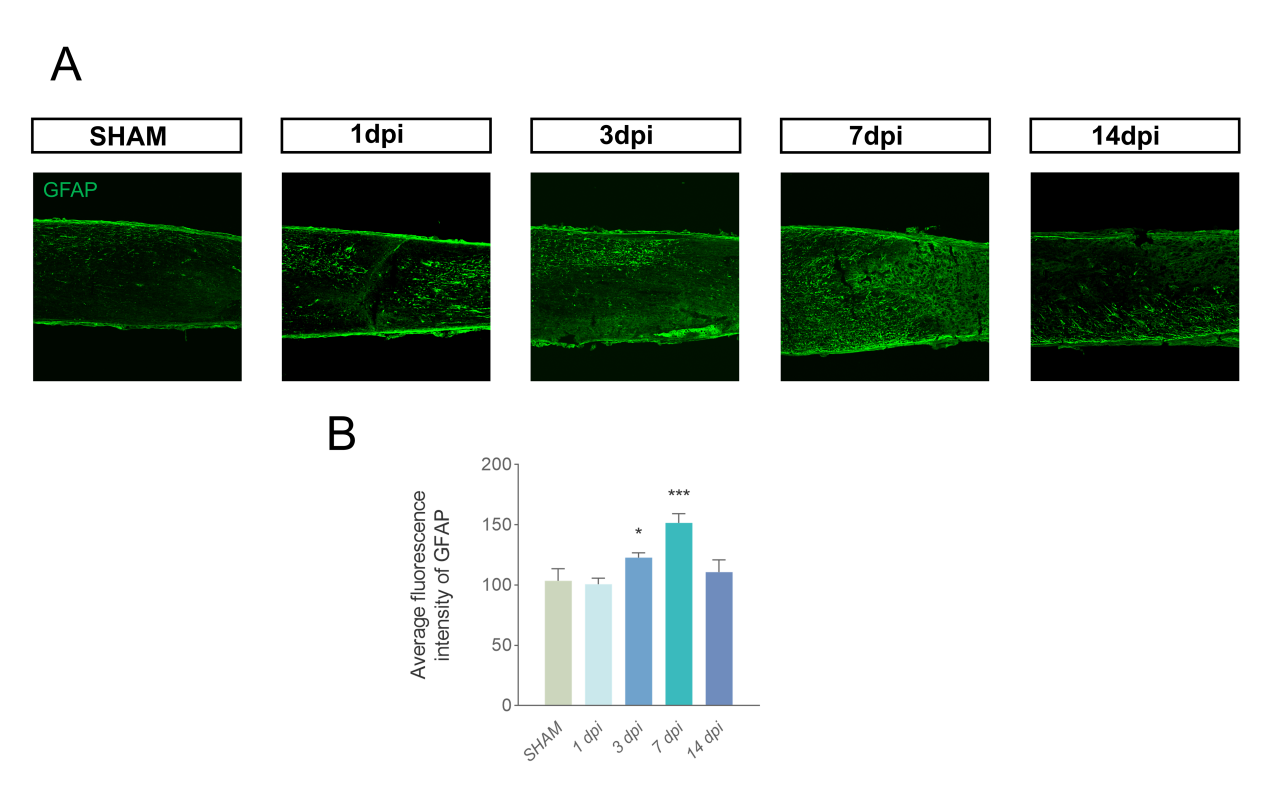


SFig 4 Detection of GFAP expression in optic nerve by IF. A) Representative images of GFAP (green) IF staining around the optic nerve injury site after ONC.Scale bar=200 µm. B)The quantitative analysis shows that Average fluorescence intensity of GFAP increased significantly at 3 dpi and 7dpi. Data are presented as the mean values of three independent experiments (mean ± SEM, n = 3 mice per group). *P < 0.05 and ***P < 0.001 vs. the SHAM group.


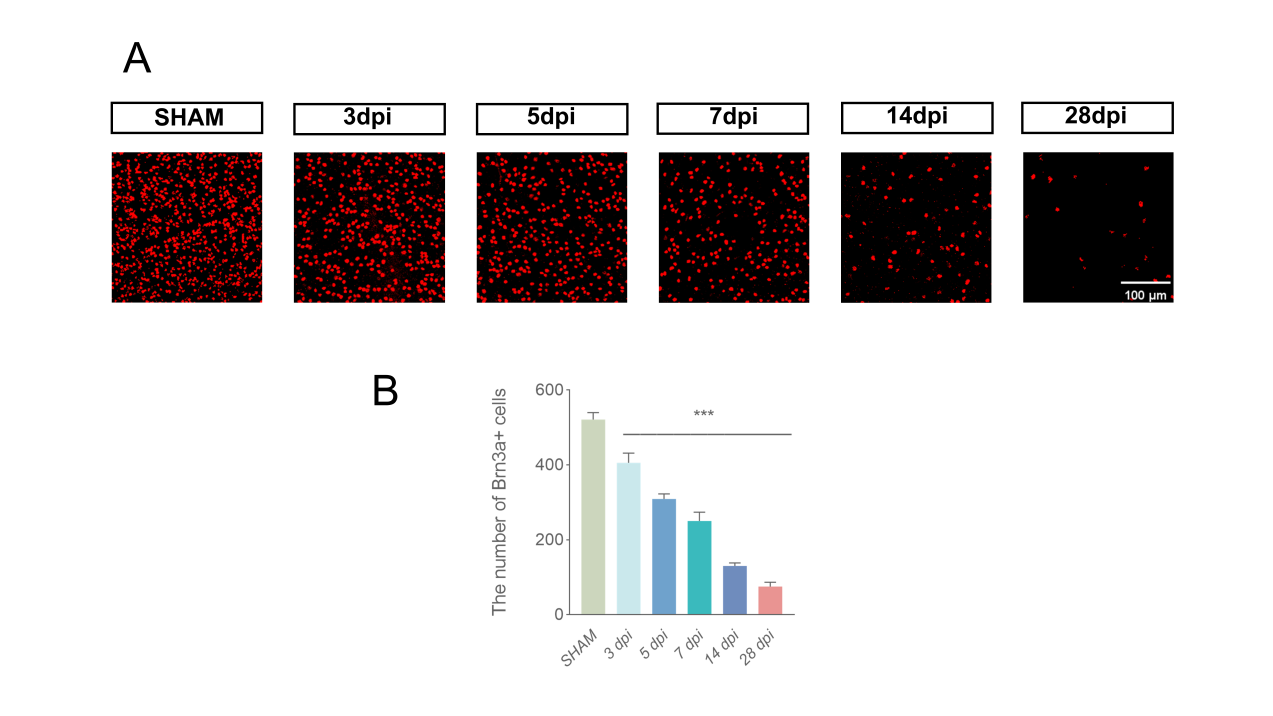


SFig 5 Detection of RGCs number in retina after optic nerve by IF. A)Representative IF maps of BRN3A in the periphery. Scale bar = 100 μm. C - D) Counts of BRN3A-positive cells in the ONC retina shows that RGCs decreased significantly at 3 dpi. Data are presented as the mean values of three independent experiments (mean ± SEM, n = 3 mice per group). ***P < 0.001 vs. the CON group.
