# Supplementary material for: Effect of astrocyte GPER on the optic nerve inflammatory response following optic nerve injury in mice
Source: Heliyon. 2024 Apr 10;10(8):e29428. doi: 10.1016/j.heliyon.2024.e29428 (PMC11024623; doi:10.1016/j.heliyon.2024.e29428)

figures 1C

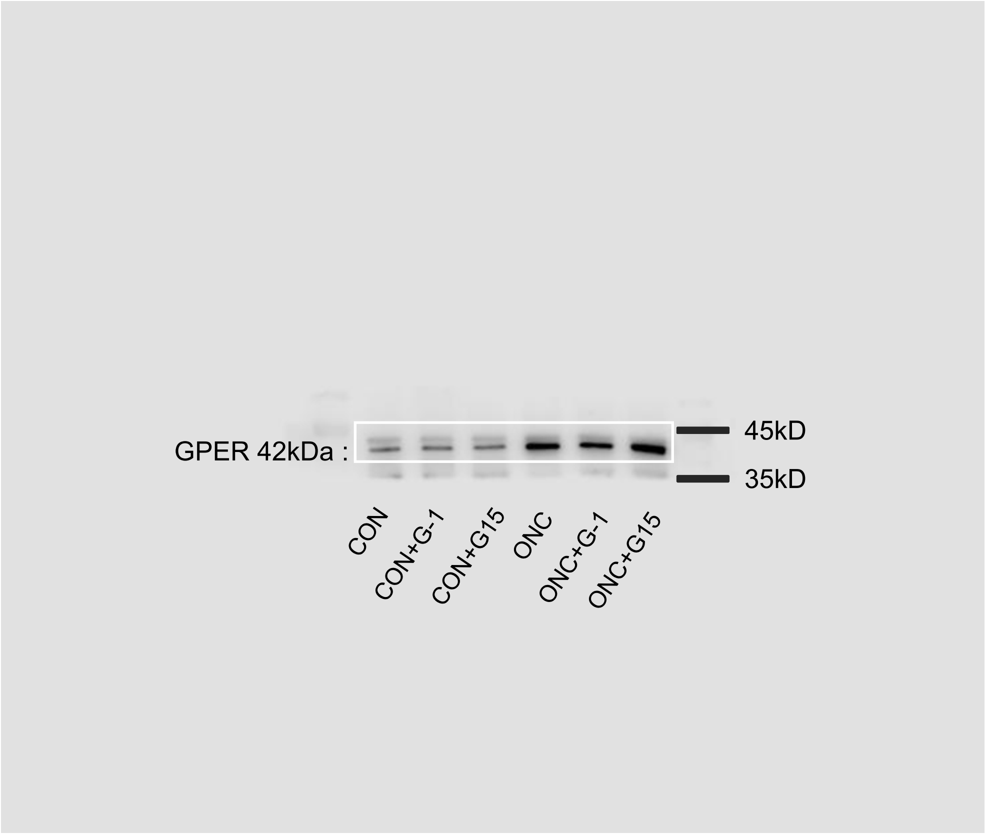
GPER 42kDa :
45kD
35kD
CON
CON+G-1
CON+G15
ONC
ONC+G-1
ONC+G15

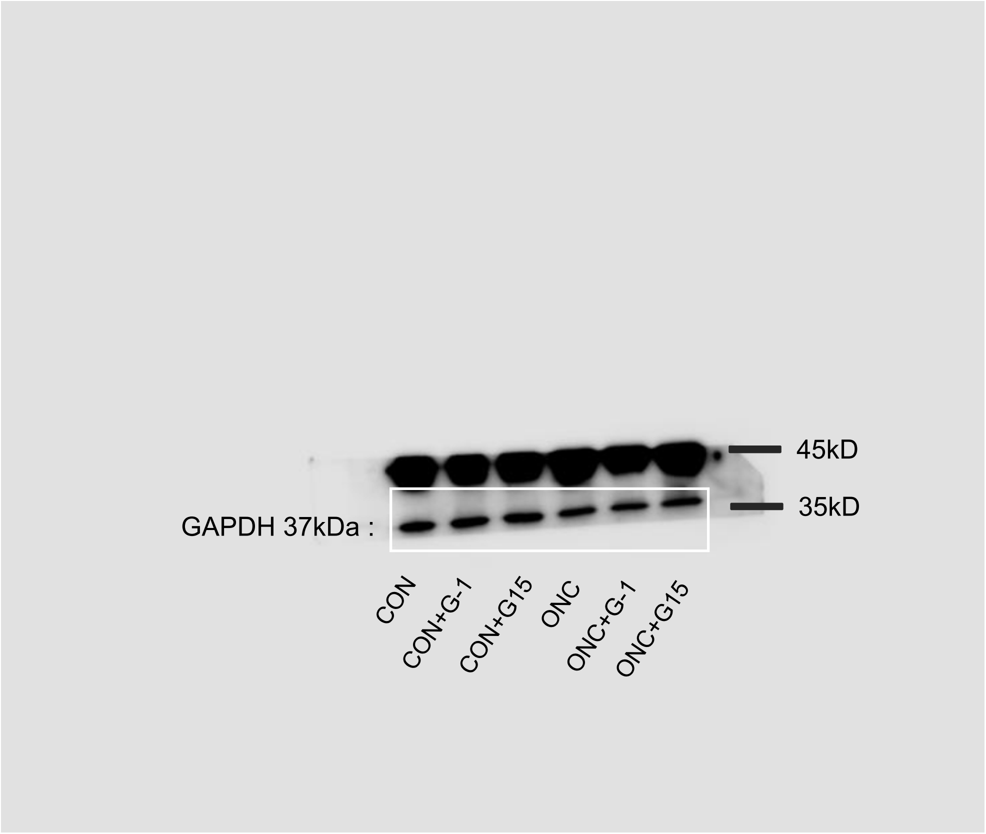
45kD
35kD
GAPDH 37kDa :
CON
CON+G-1
CON+G15
ONC
ONC+G-1
ONC+G15

figures 1F

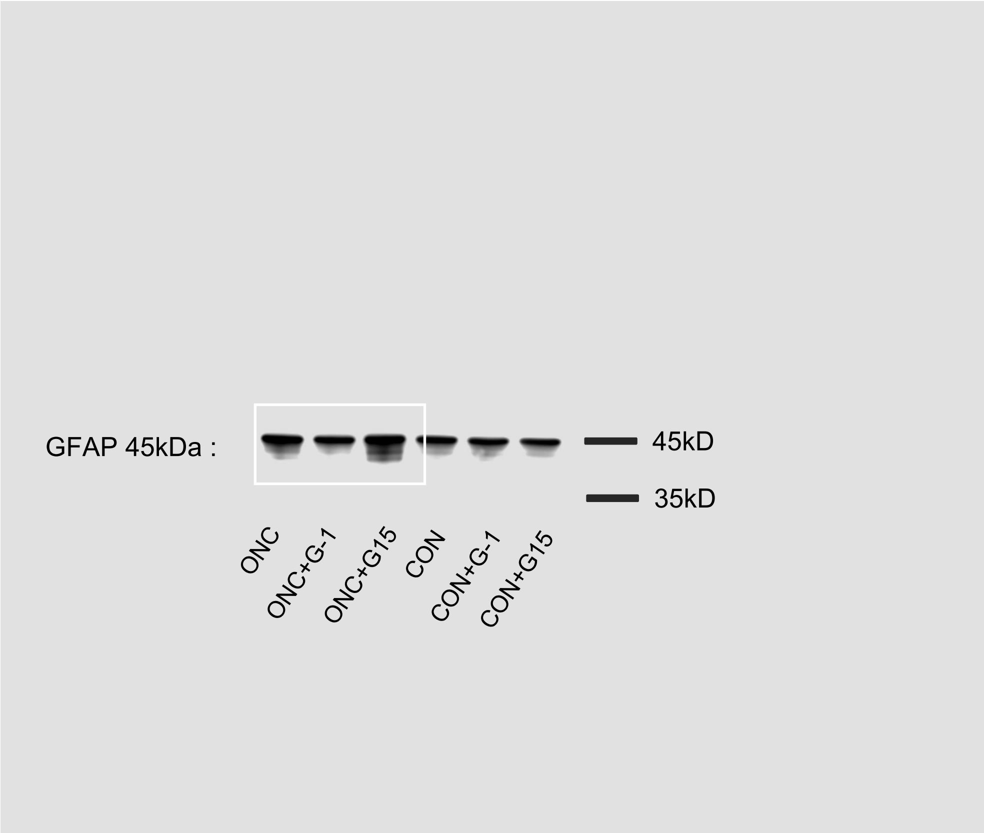
GFAP 45kDa :
45kD
35kD
ONC
ONC+G-1
ONC+G15
CON
CON+G-1
CON+G15

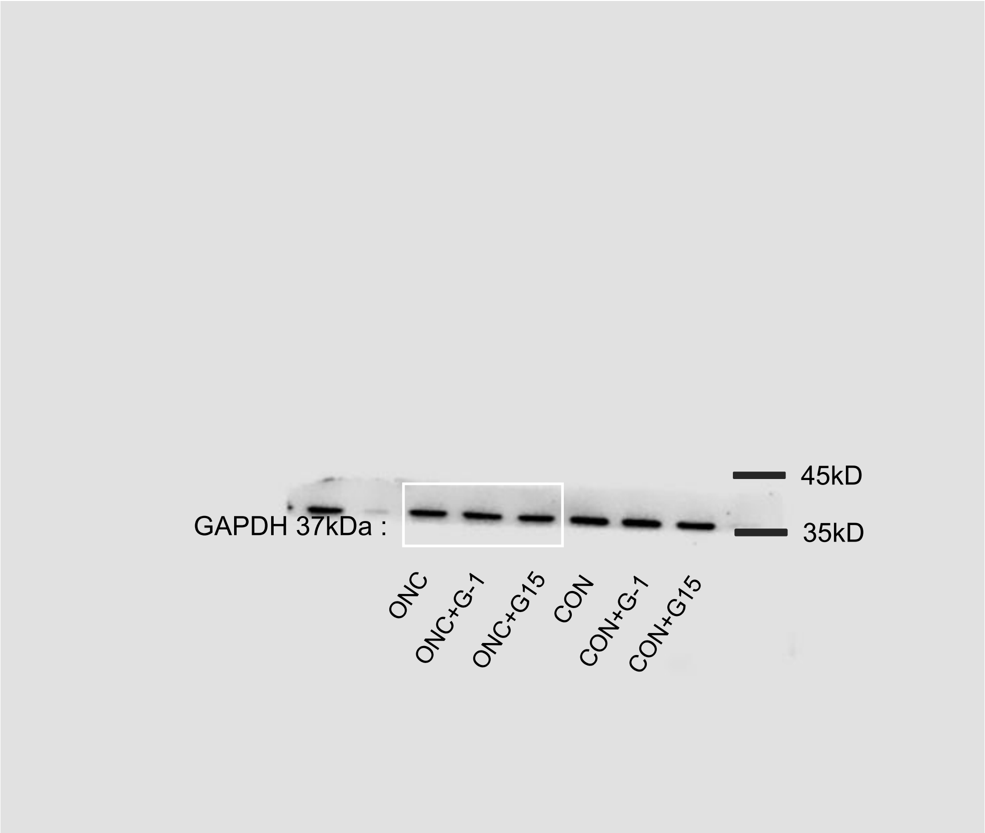
GAPDH 37kDa :
45kD
35kD
ONC
ONC+G-1
ONC+G15
CON
CON+G-1
CON+G15

figures 2A

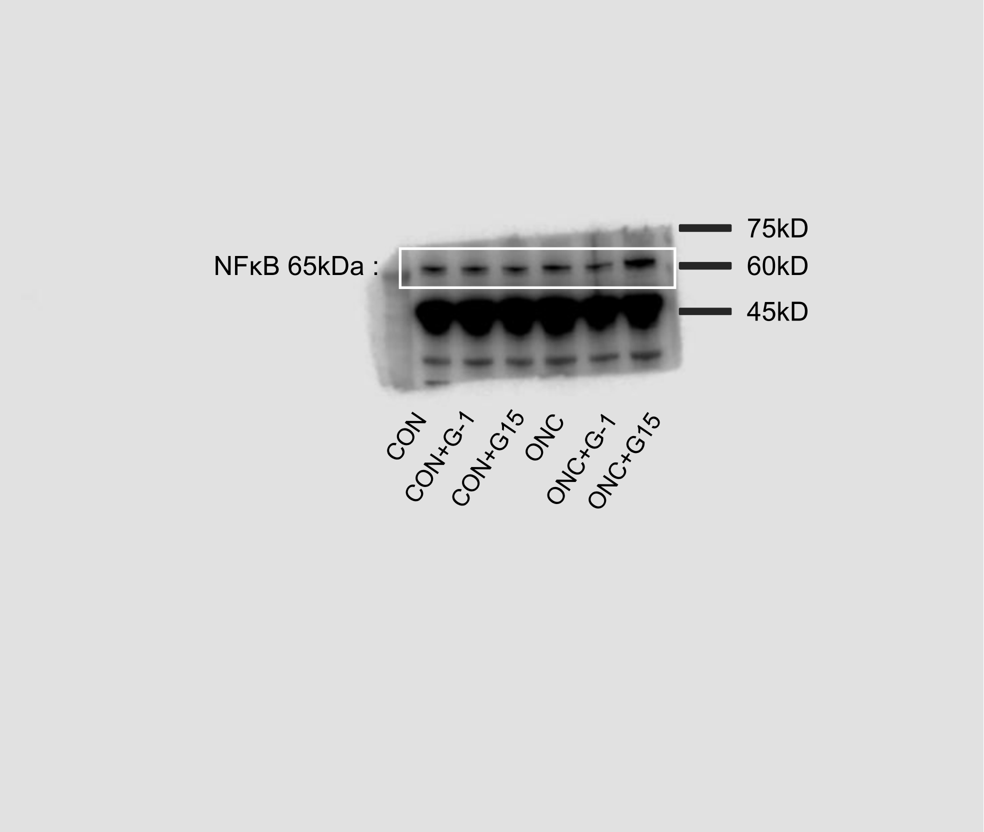
NFκB 65kDa :
75kD
60kD
45kD
CON
CON+G-1
CON+G15
ONC
ONC+G-1
ONC+G15

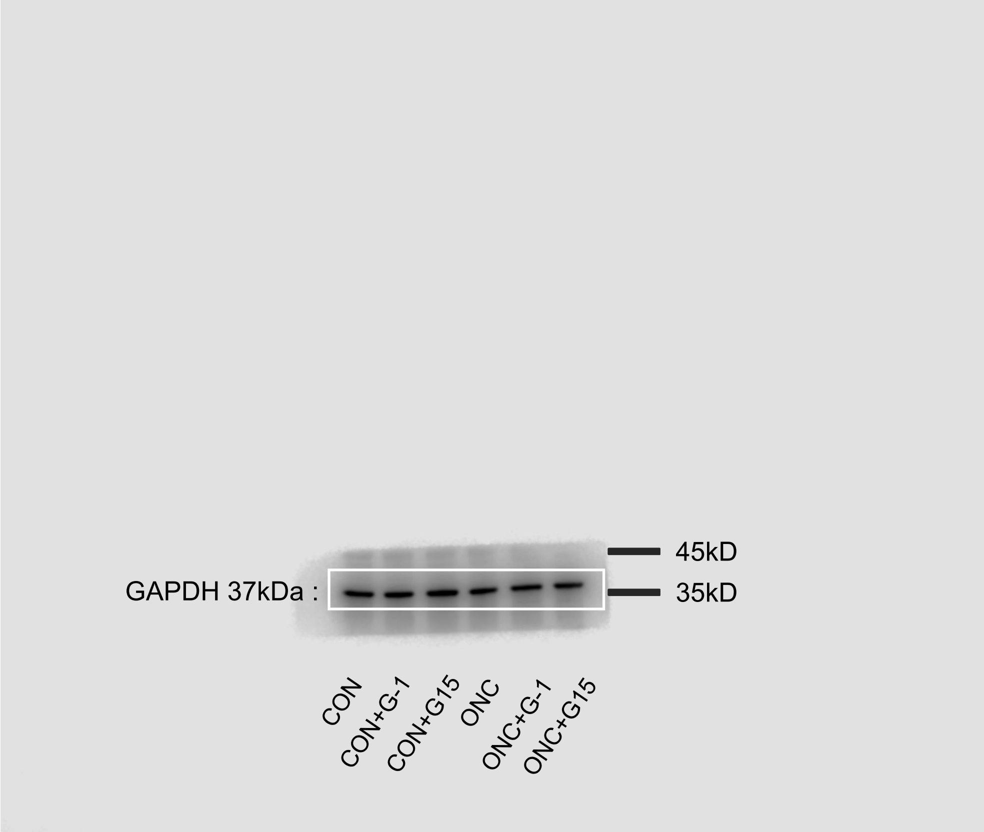
GAPDH 37kDa :
45kD
35kD
CON
CON+G-1
CON+G15
ONC
ONC+G-1
ONC+G15

figures 2C

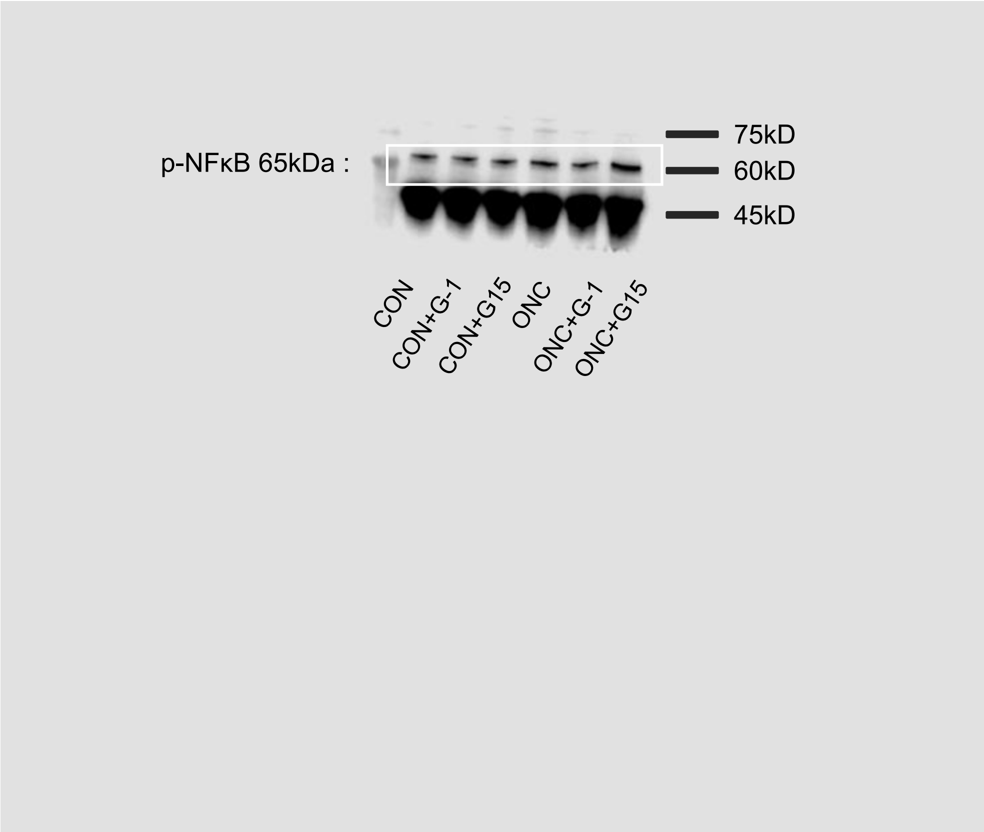
p-NFκB 65kDa :
75kD
60kD
45kD
CON
CON+G-1
CON+G15
ONC
ONC+G-1
ONC+G15

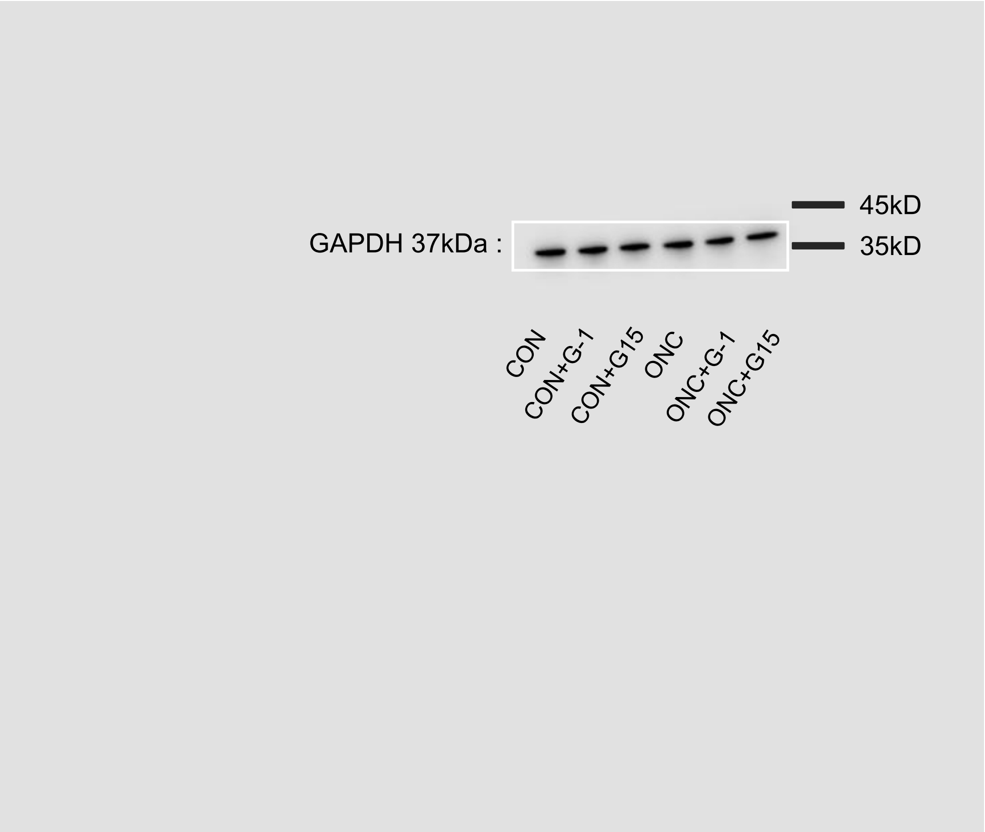
GAPDH 37kDa :
45kD
35kD
CON
CON+G-1
CON+G15
ONC
ONC+G-1
ONC+G15

figures 2E

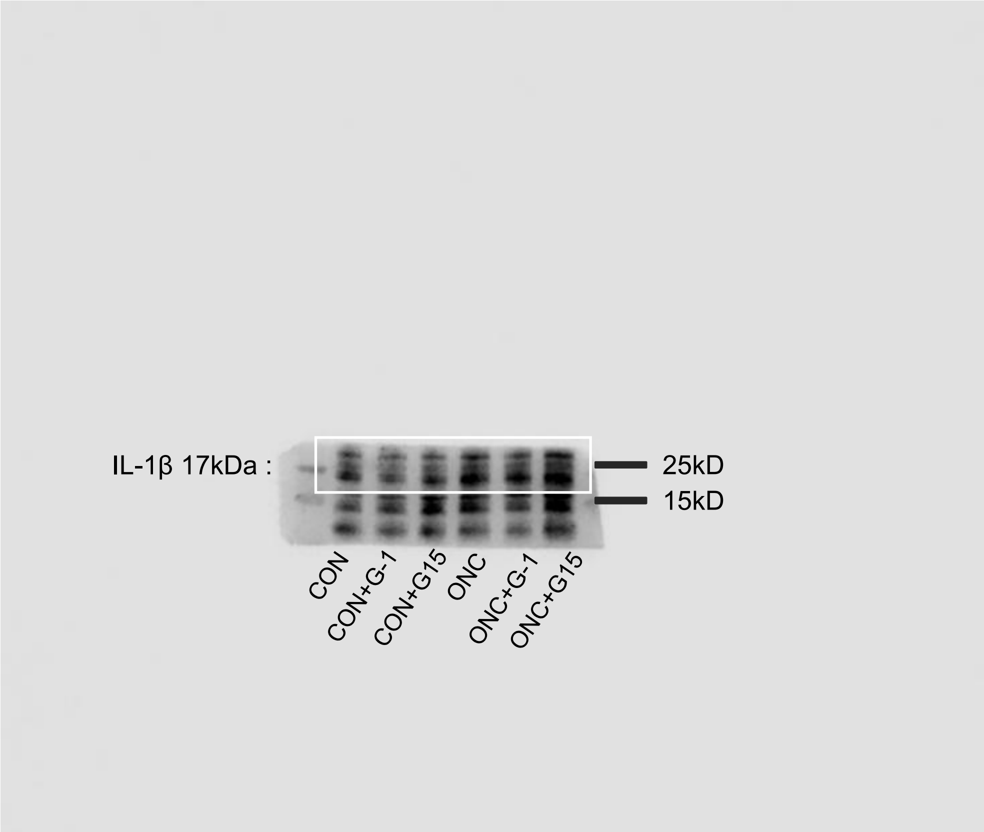
IL-1β 17kDa :
25kD
15kD
CON
CON+G-1
CON+G15
ONC
ONC+G-1
ONC+G15

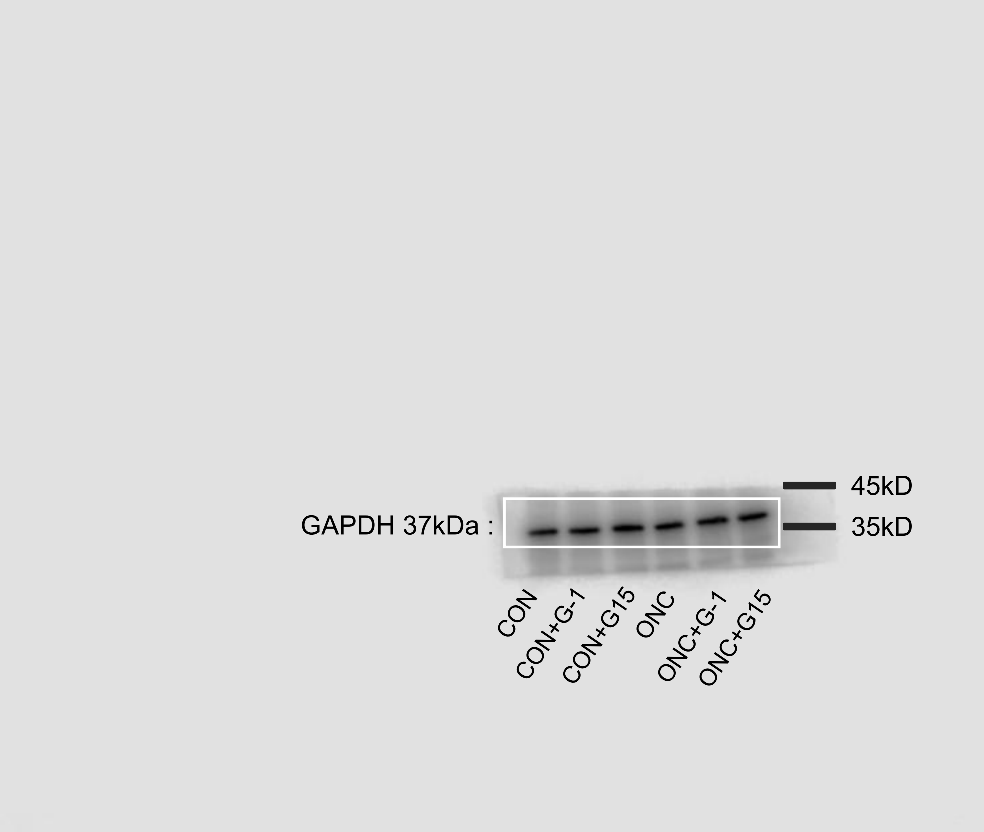
45kD
GAPDH 37kDa :
35kD
CON
CON+G-1
CON+G15
ONC
ONC+G-1
ONC+G15

figures 2G

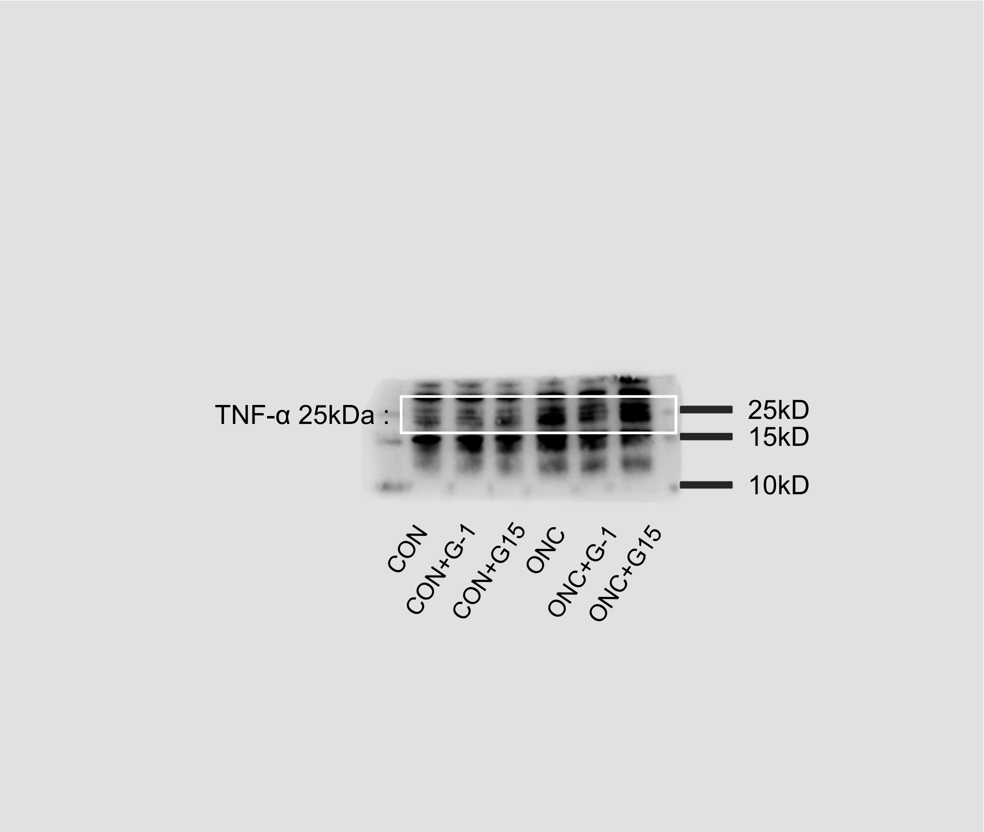
TNF-α 25kDa :
25kD
15kD
10kD
CON
CON+G-1
CON+G15
ONC
ONC+G-1
ONC+G15

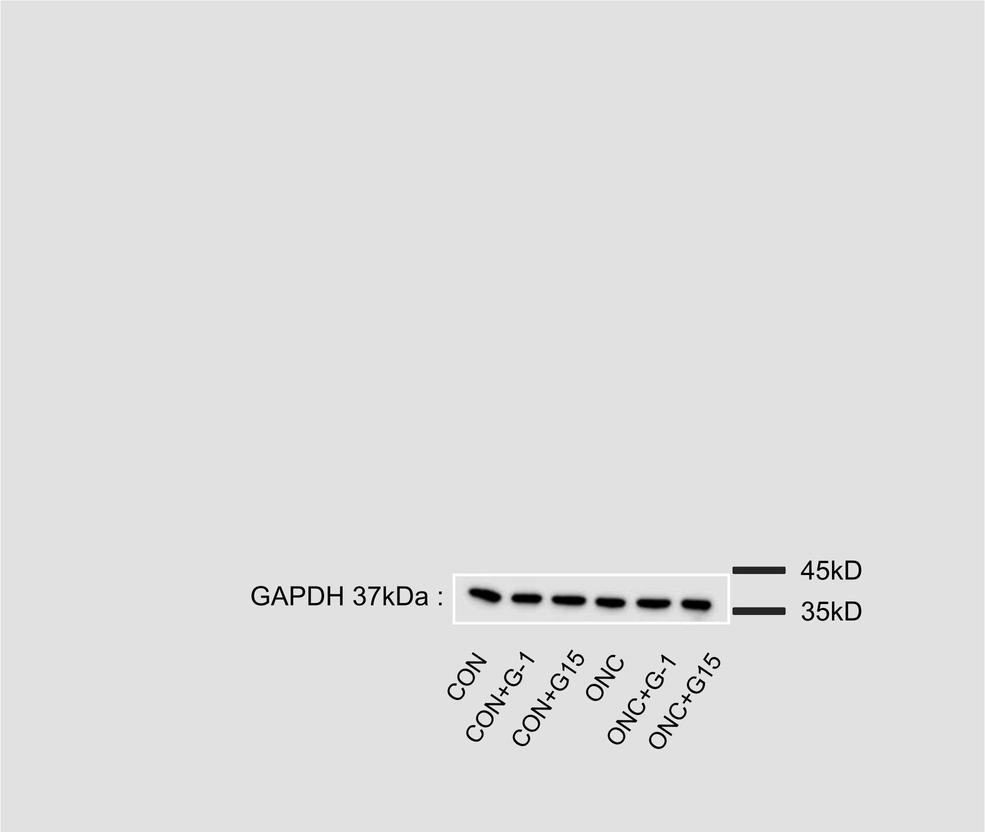
GAPDH 37kDa :
45kD
35kD
CON
CON+G-1
CON+G15
ONC
ONC+G-1
ONC+G15

figures 4D

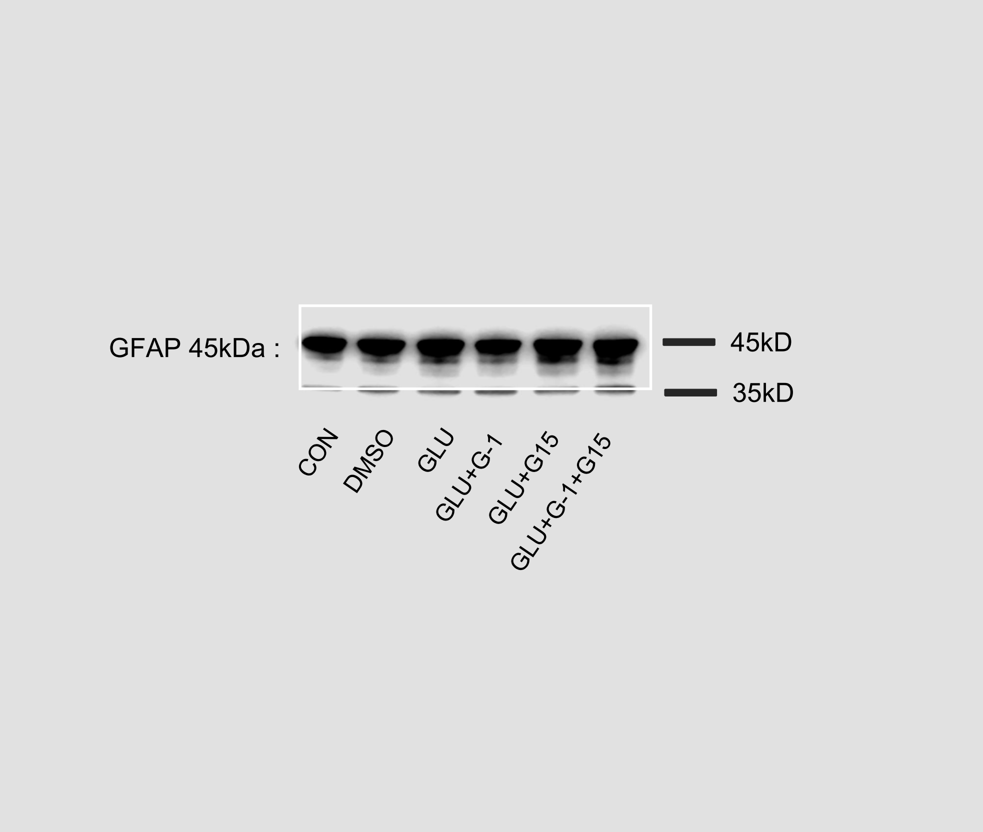
GFAP 45kDa :
45kD
35kD
CON
DMSO
GLU
GLU+G-1
GLU+G15
GLU+G-1+G15

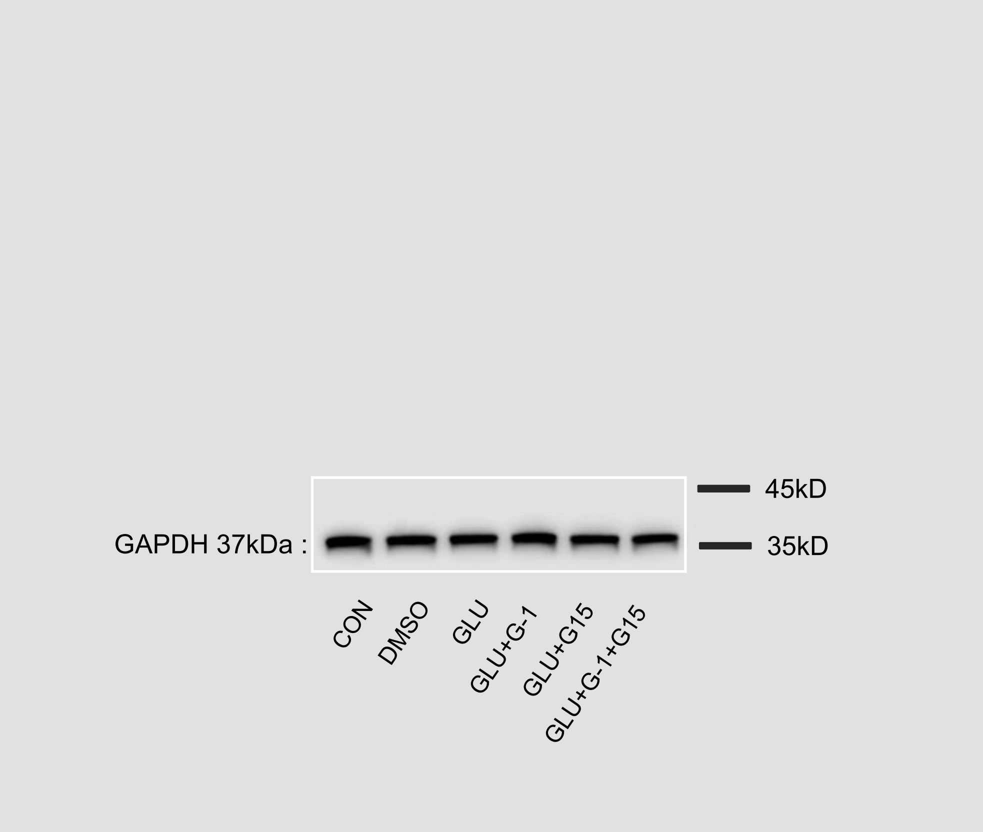
GAPDH 37kDa :
45kD
35kD
CON
DMSO
GLU
GLU+G-1
GLU+G15
GLU+G-1+G15

figures 5A

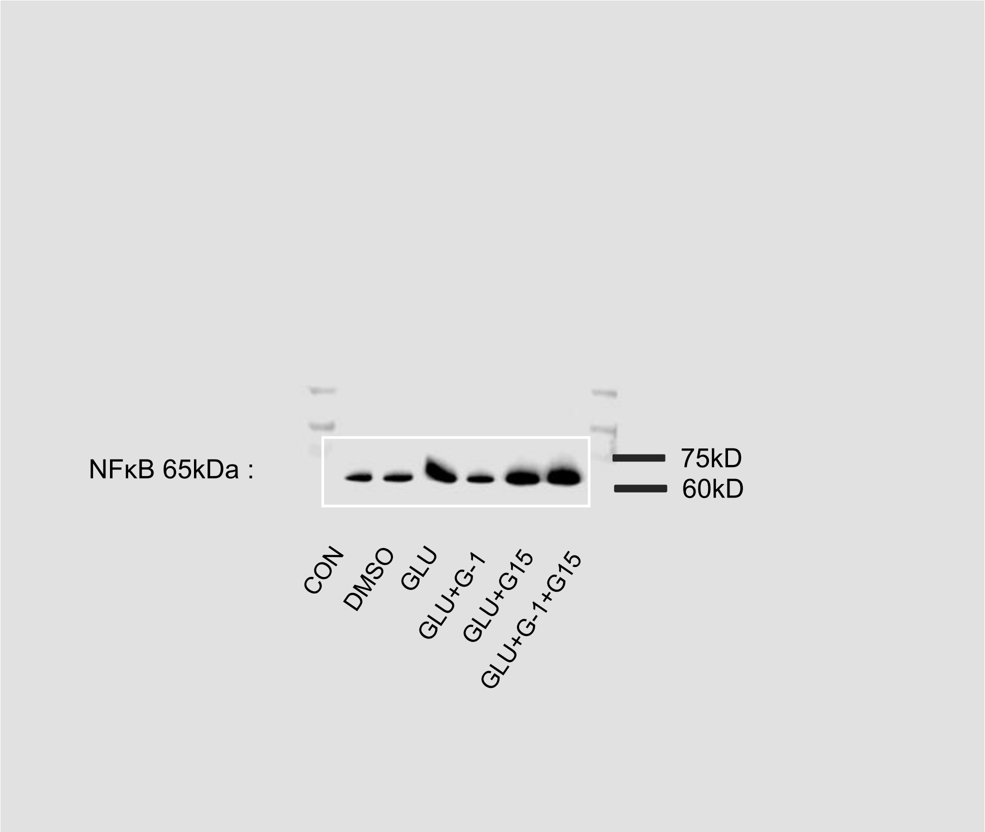

NFκB 65kDa :
75kD
60kD
CON
DMSO
GLU
GLU+G-1
GLU+G15
GLU+G-1+G15

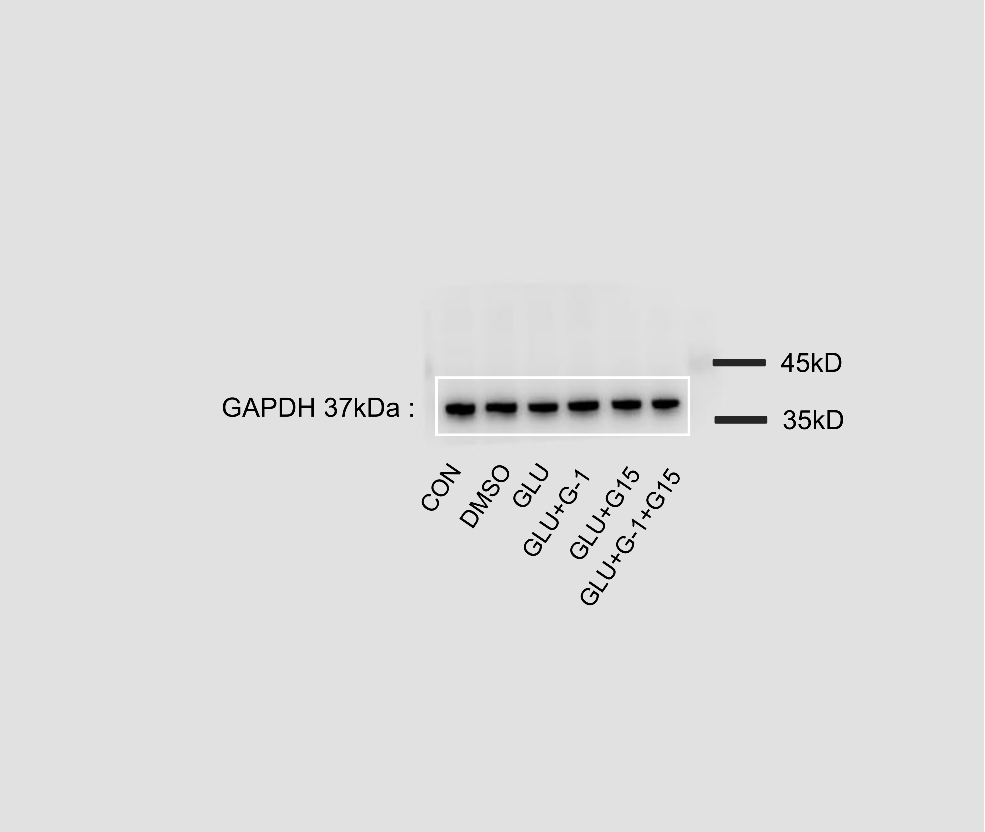

GAPDH 37kDa :
45kD
35kD
CON
DMSO
GLU
GLU+G-1
GLU+G15
GLU+G-1+G15

figures 5C

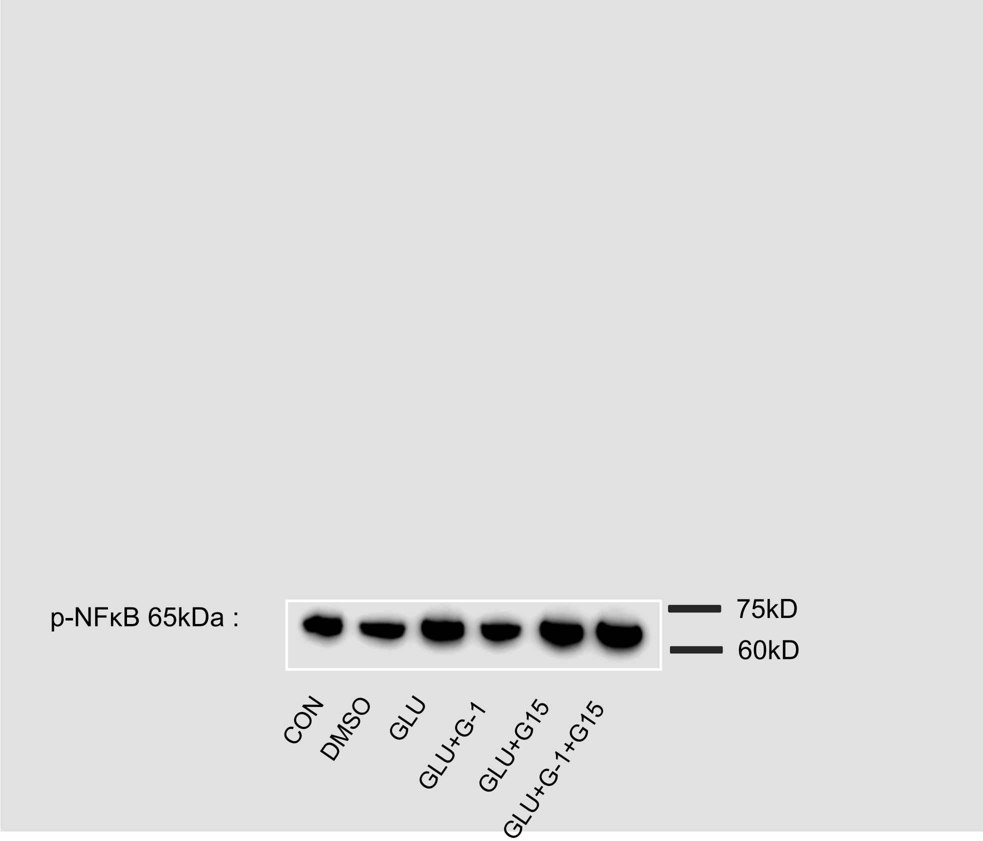
p-NFκB 65kDa :
75kD
60kD
CON
DMSO
GLU
GLU+G-1
GLU+G15
GLU+G-1+G15

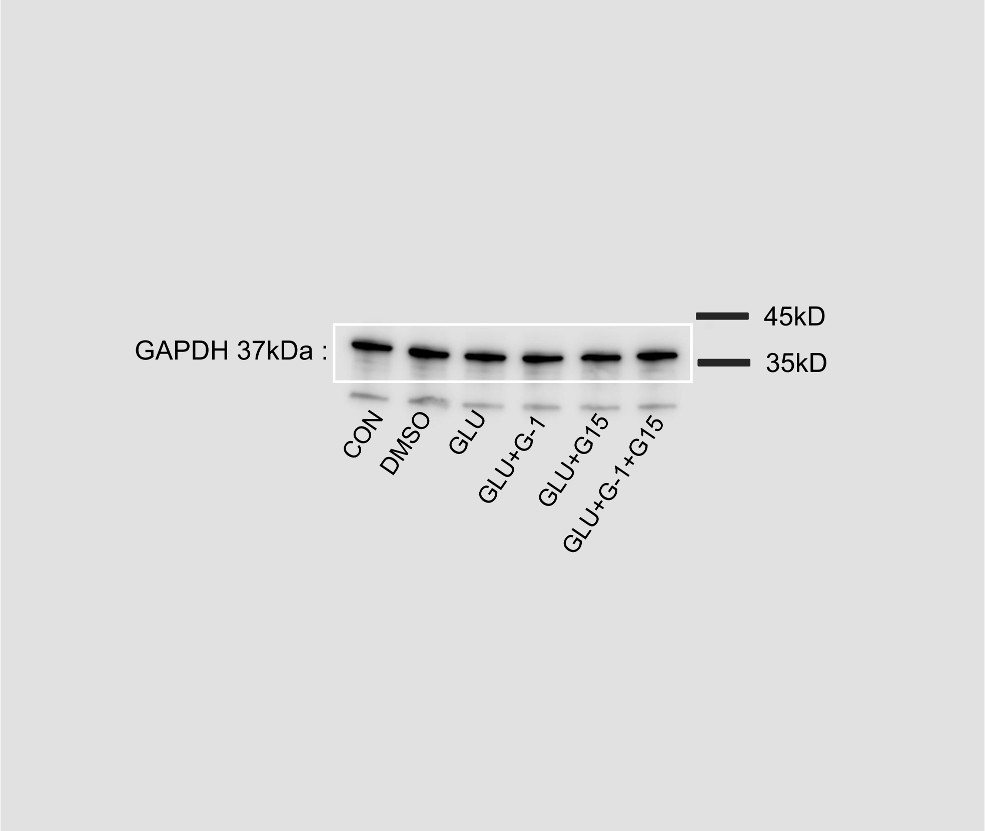
GAPDH 37kDa :
45kD
35kD
CON
DMSO
GLU
GLU+G-1
GLU+G15
GLU+G-1+G15

figures 5E

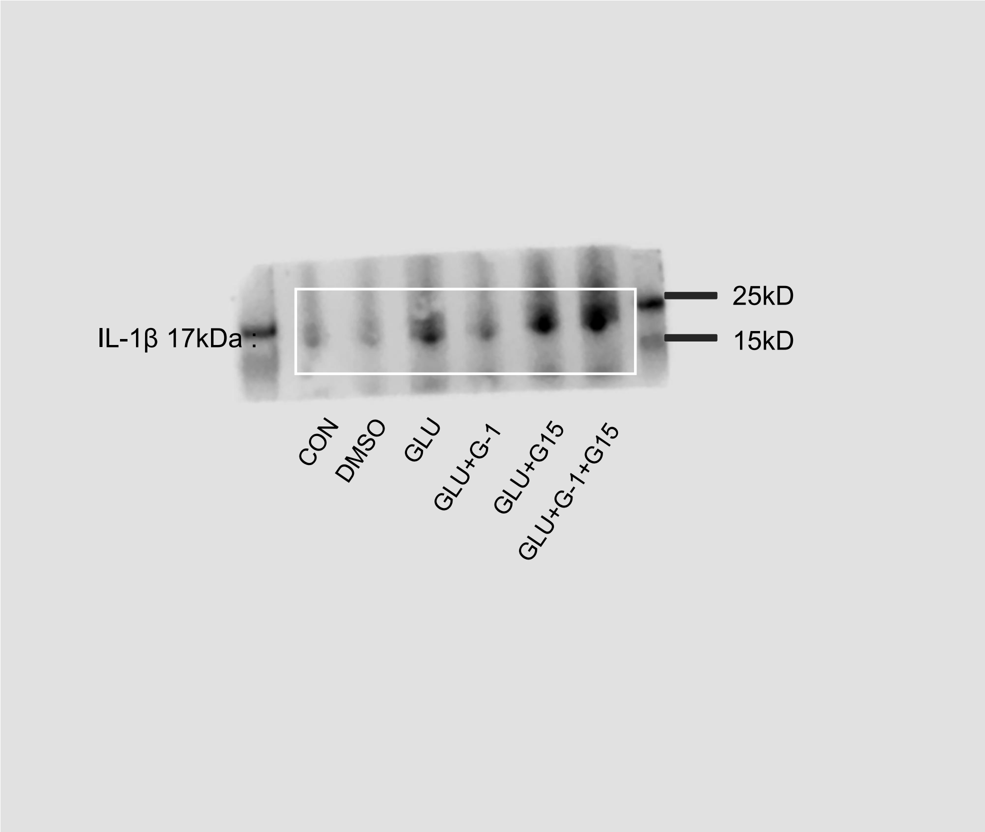
IL-1β 17kDa :
25kD
15kD
CON
DMSO
GLU
GLU+G-1
GLU+G15
GLU+G-1+G15

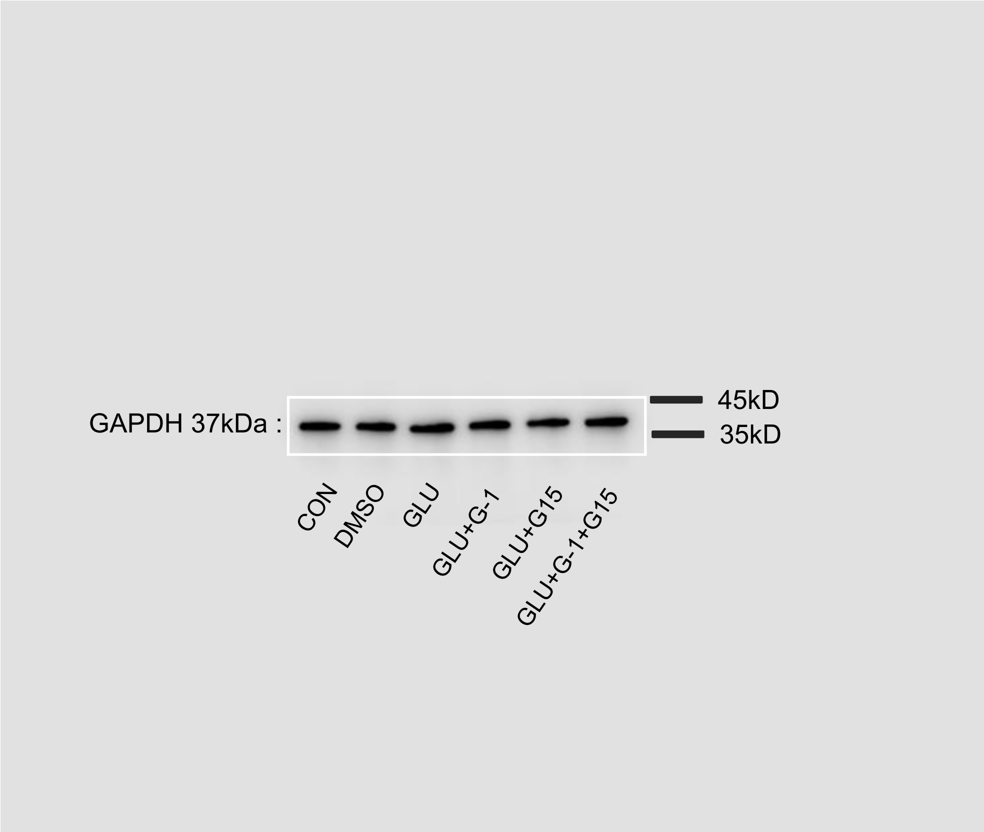
GAPDH 37kDa :
45kD
35kD
CON
DMSO
GLU
GLU+G-1
GLU+G15
GLU+G-1+G15

figures 5G

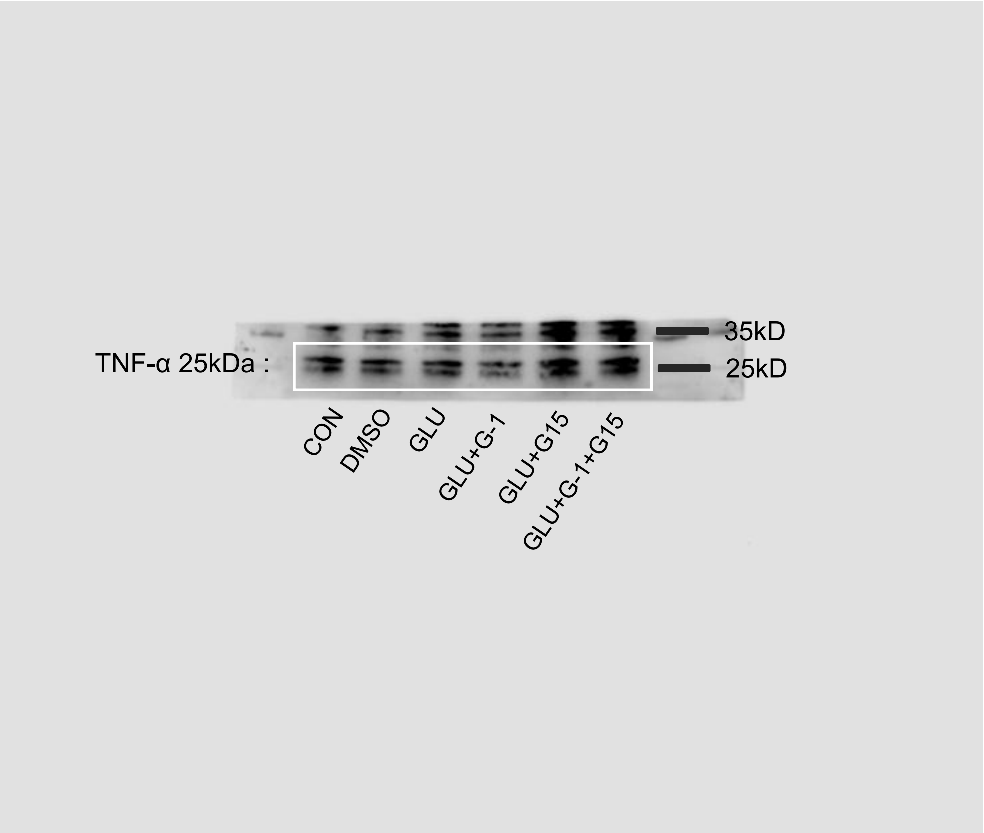

TNF-α 25kDa :
35kD
25kD
CON
DMSO
GLU
GLU+G-1
GLU+G15
GLU+G-1+G15

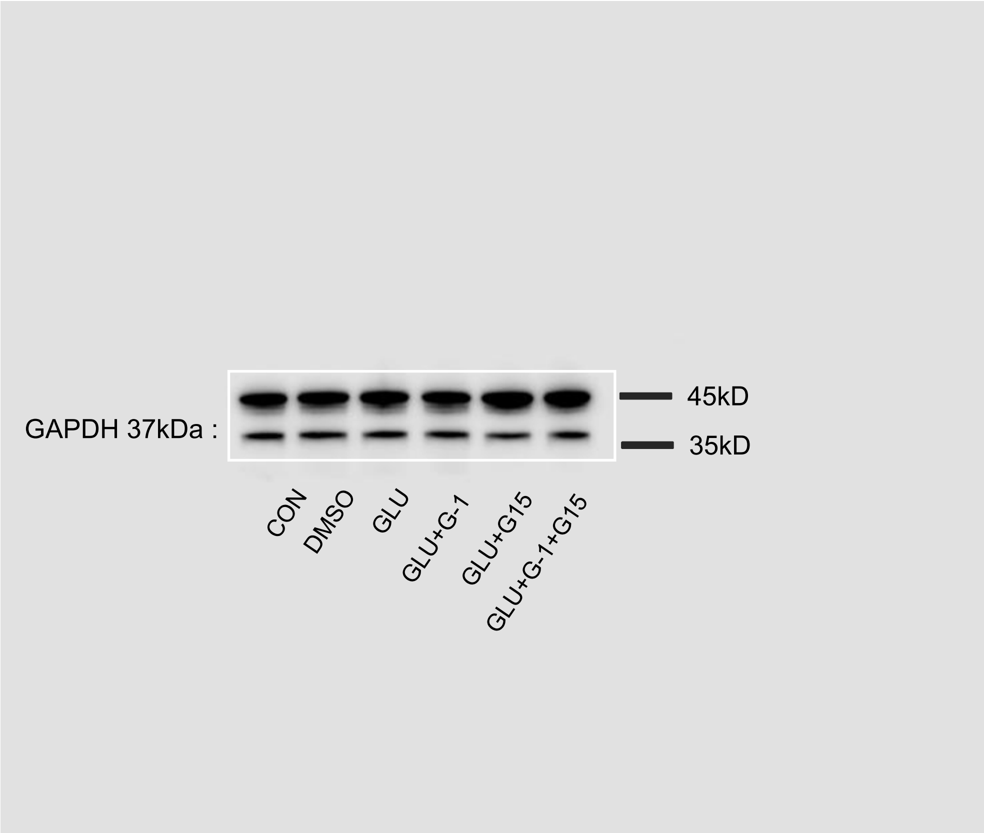

GAPDH 37kDa :
45kD
35kD
CON
DMSO
GLU
GLU+G-1
GLU+G15
GLU+G-1+G15

figures 6A

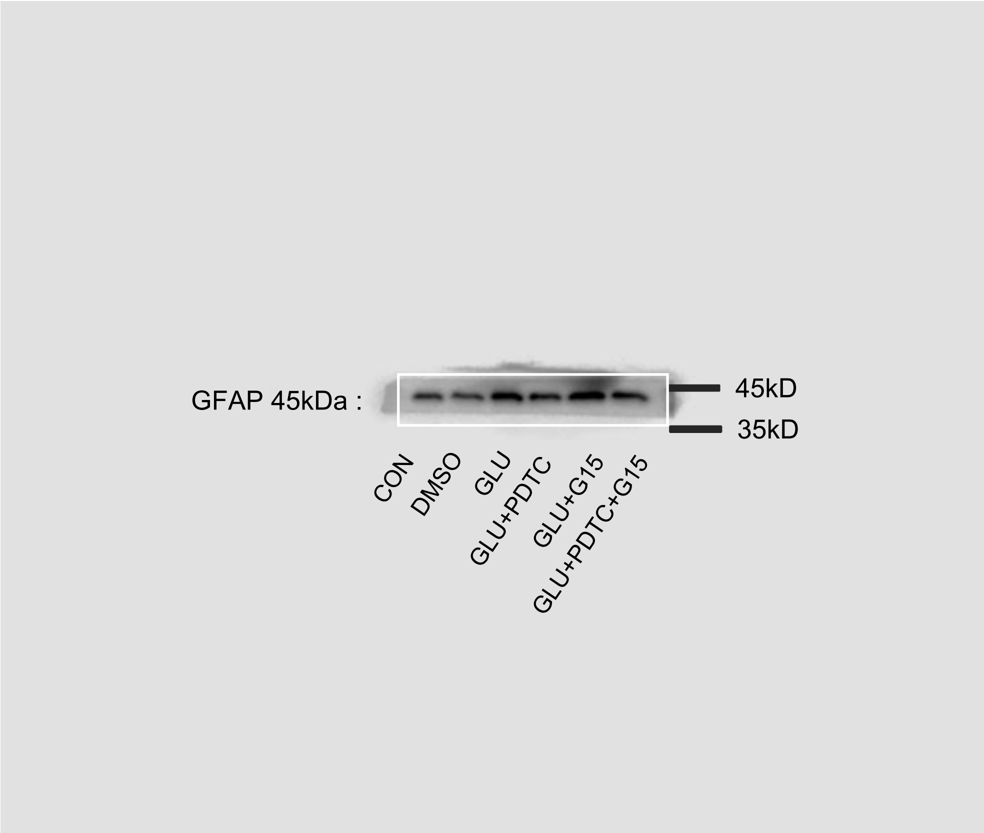
GFAP 45kDa :
45kD
35kD
CON
DMSO
GLU
GLU+PDTC
GLU+G15
GLU+PDTC+G15

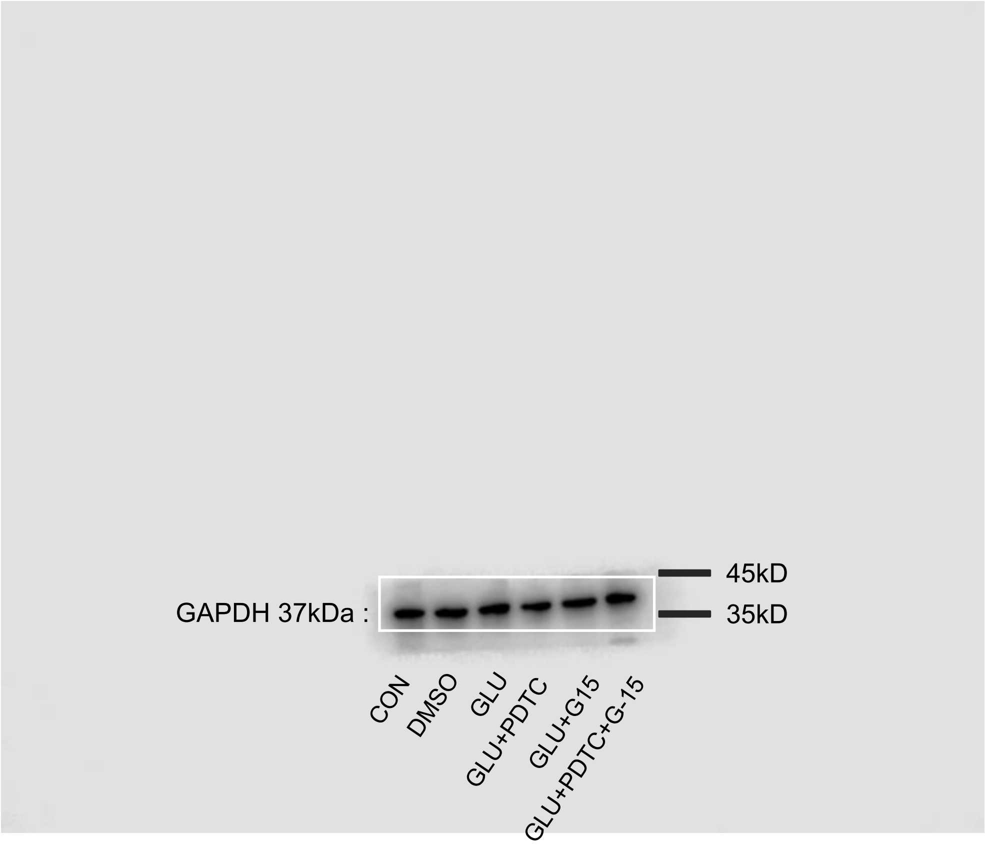
GAPDH 37kDa :
45kD
35kD
CON
DMSO
GLU
GLU+PDTC
GLU+G15
GLU+PDTC+G-15

figures 6A

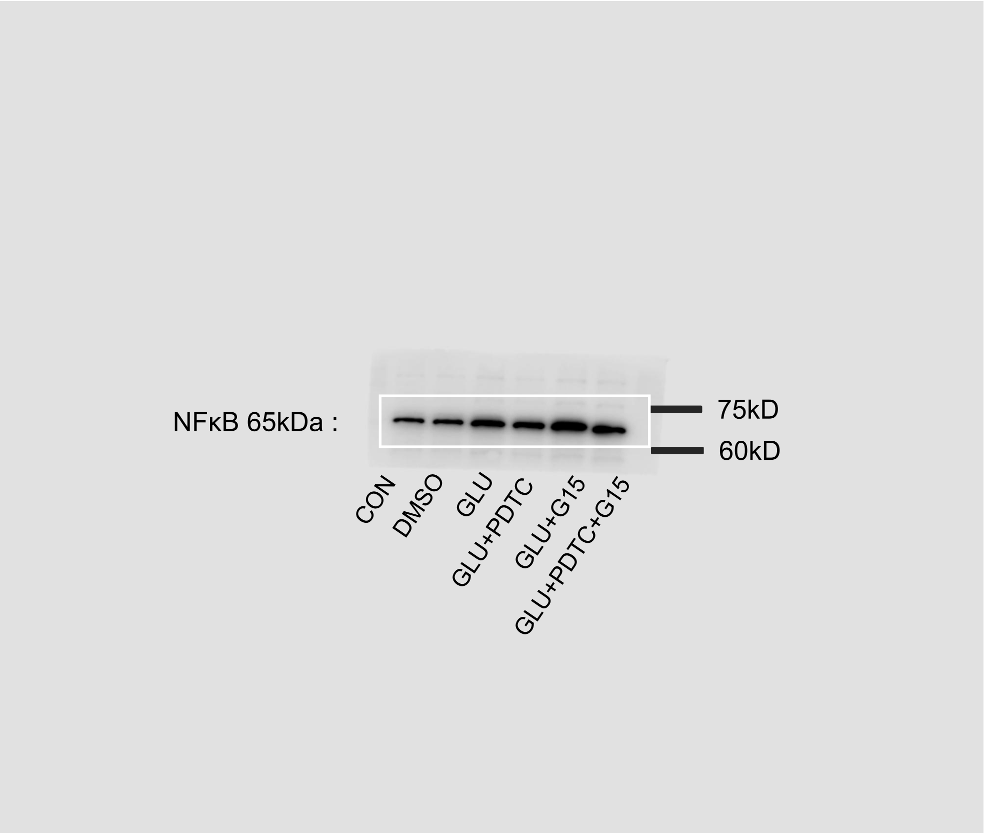
NFκB 65kDa :
75kD
60kD
CON
DMSO
GLU
GLU+PDTC
GLU+G15
GLU+PDTC+G15

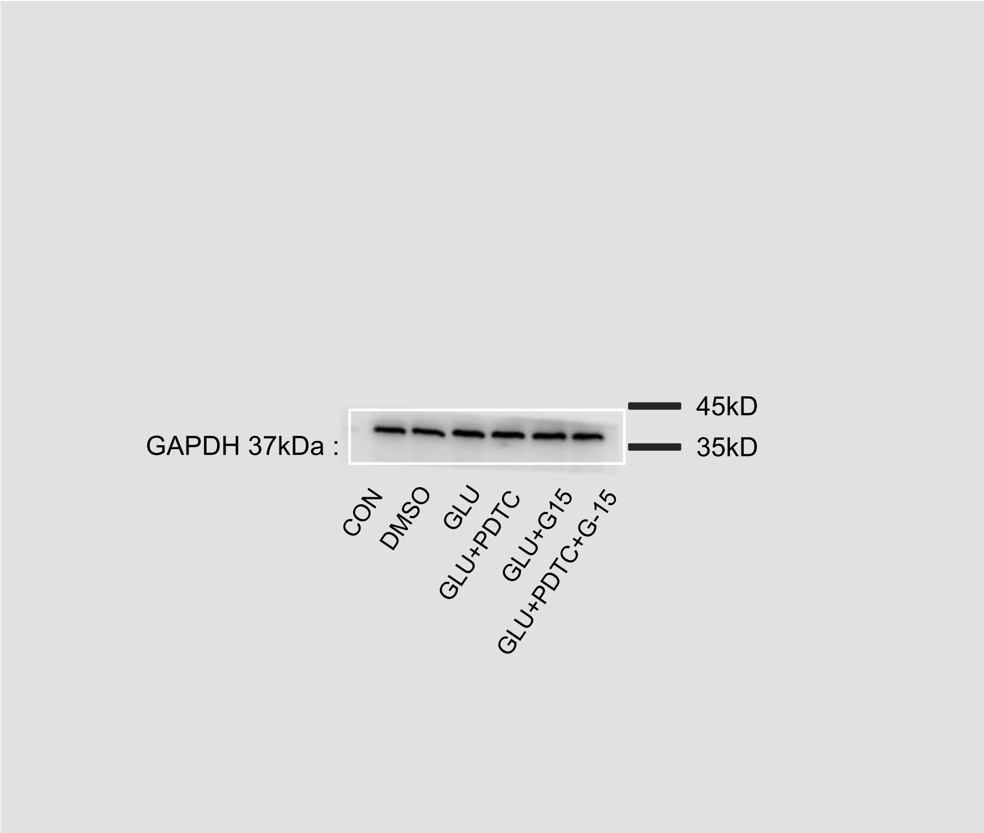
GAPDH 37kDa :
45kD
35kD
CON
DMSO
GLU
GLU+PDTC
GLU+G15
GLU+PDTC+G-15

figures 6A

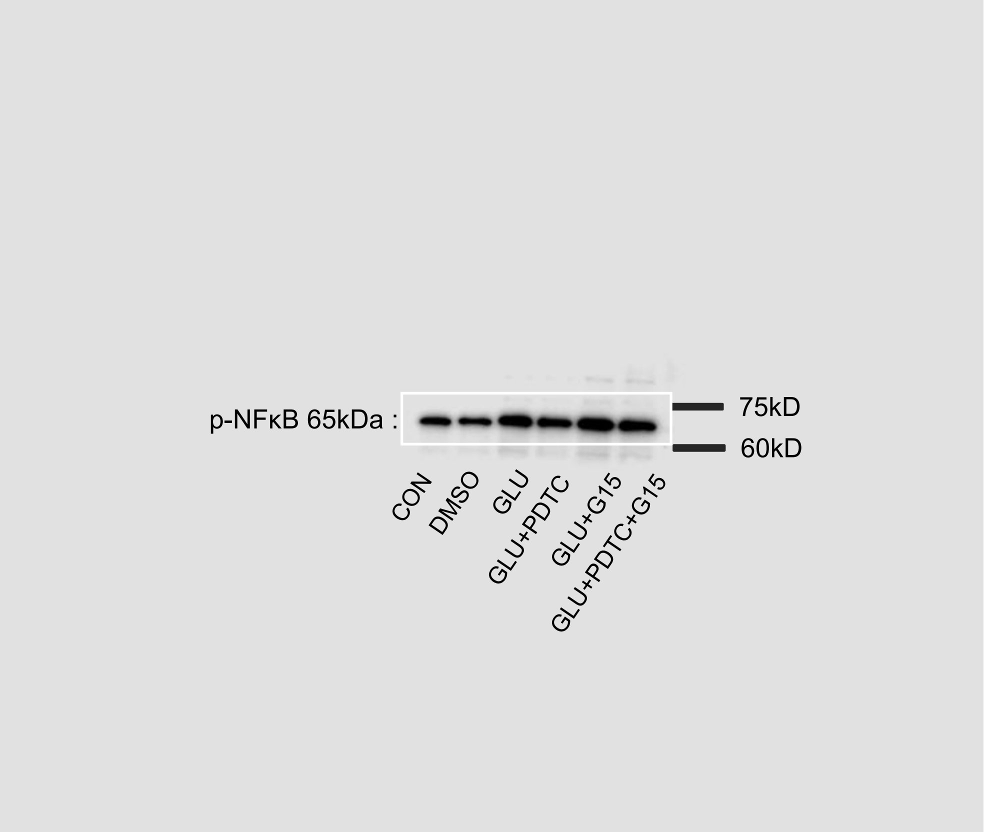
p-NFκB 65kDa :
75kD
60kD
CON
DMSO
GLU
GLU+PDTC
GLU+G15
GLU+PDTC+G15

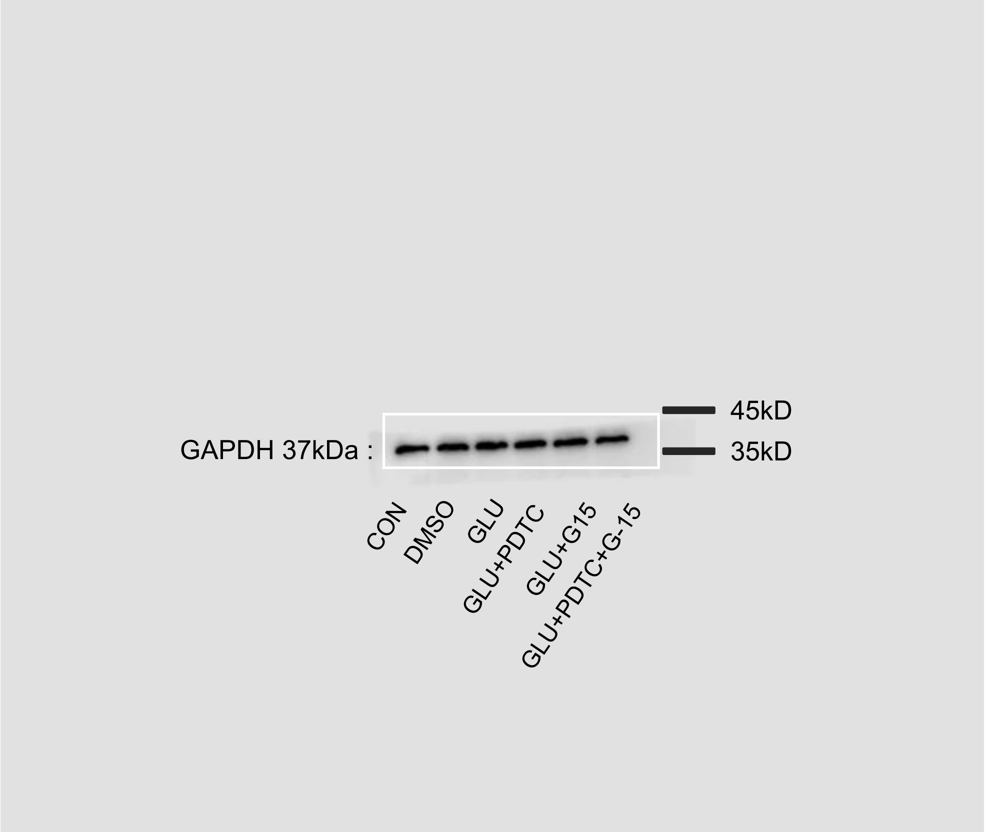
GAPDH 37kDa :
45kD
35kD
CON
DMSO
GLU
GLU+PDTC
GLU+G15
GLU+PDTC+G-15

figures 6A

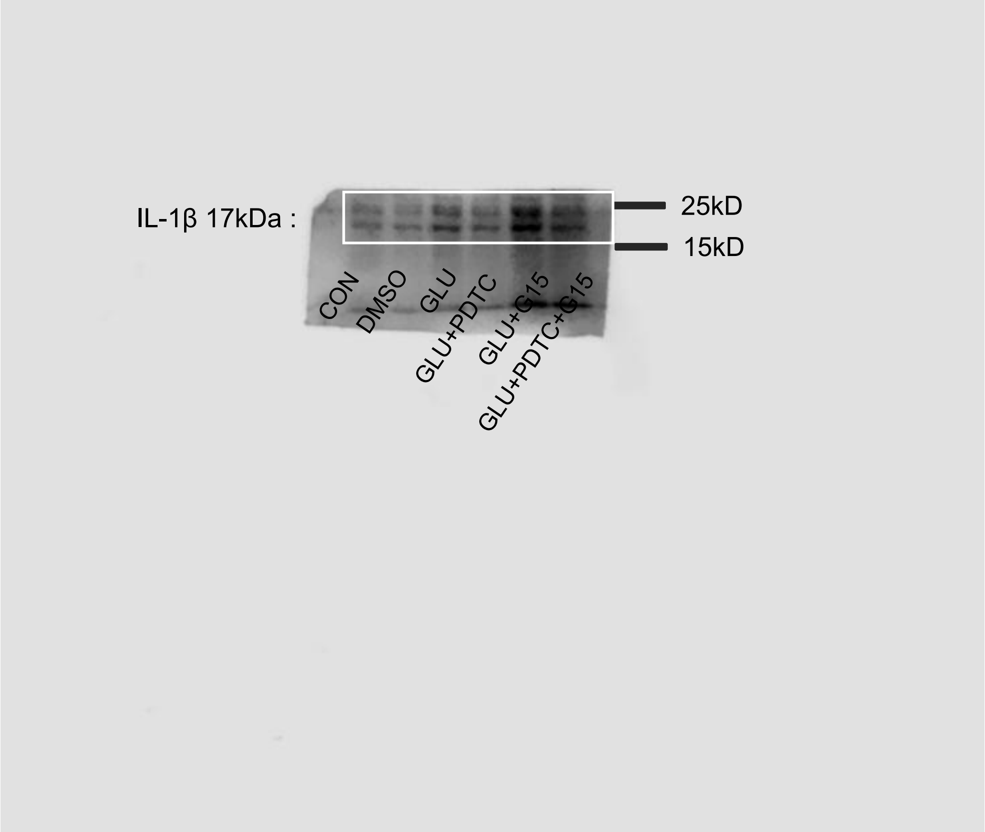
IL-1β 17kDa :
25kD
15kD
CON
DMSO
GLU
GLU+PDTC
GLU+G15
GLU+PDTC+G15

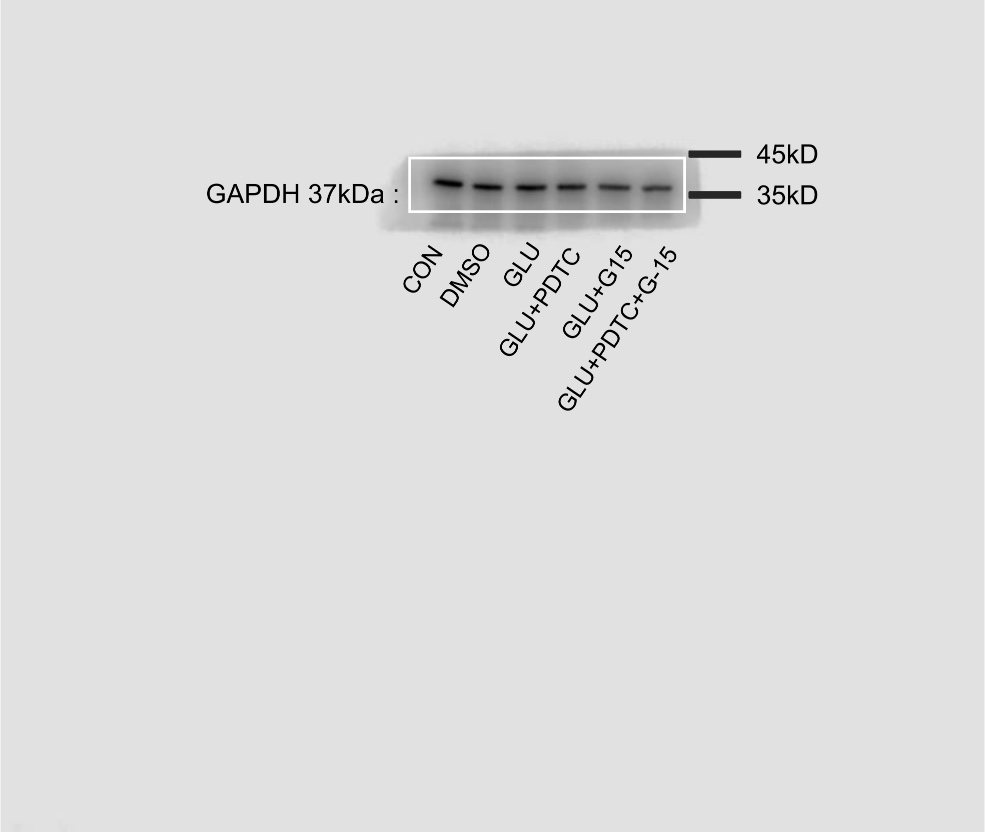
GAPDH 37kDa :
45kD
35kD
CON
DMSO
GLU
GLU+PDTC
GLU+G15
GLU+PDTC+G-15

figures 6A

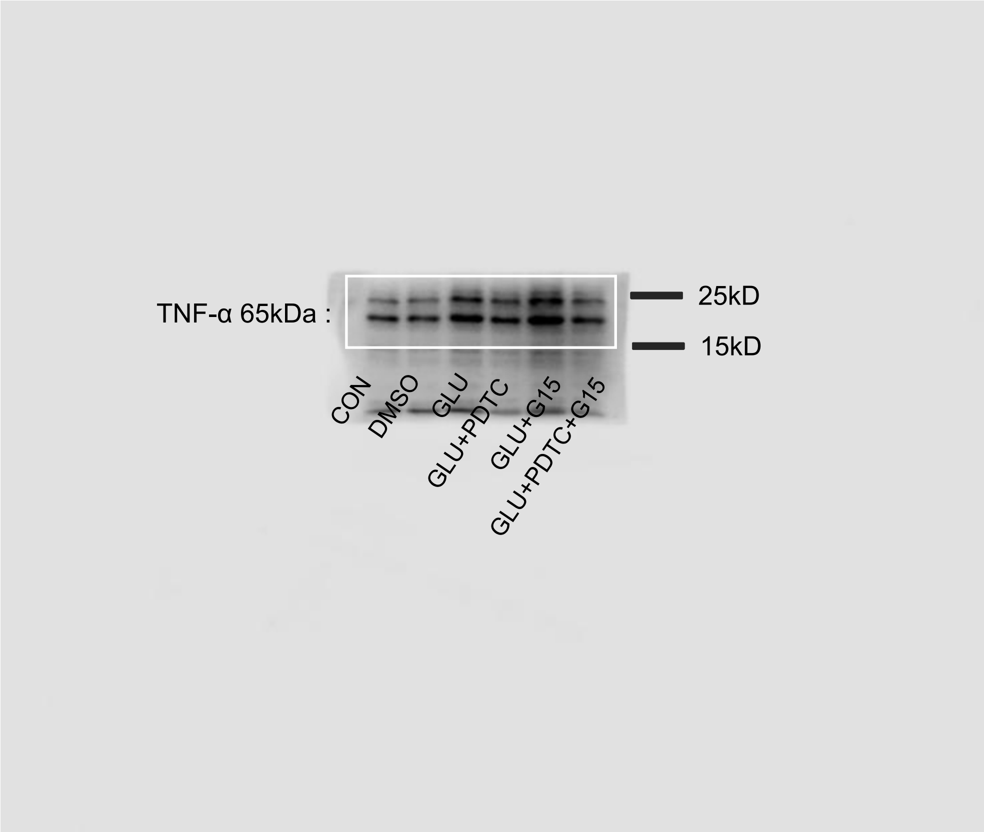

TNF-α 65kDa :
25kD
15kD
CON
DMSO
GLU
GLU+PDTC
GLU+G15
GLU+PDTC+G15

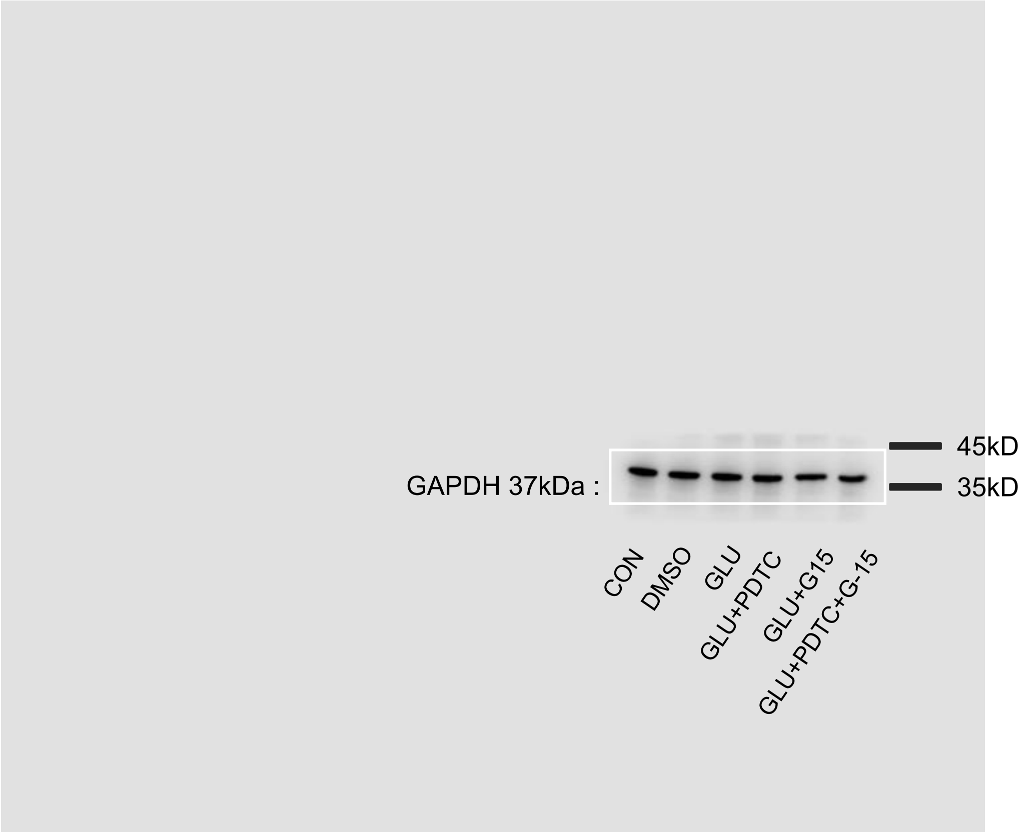

GAPDH 37kDa :
45kD
35kD
CON
DMSO
GLU
GLU+PDTC
GLU+G15
GLU+PDTC+G-15

figures 7A

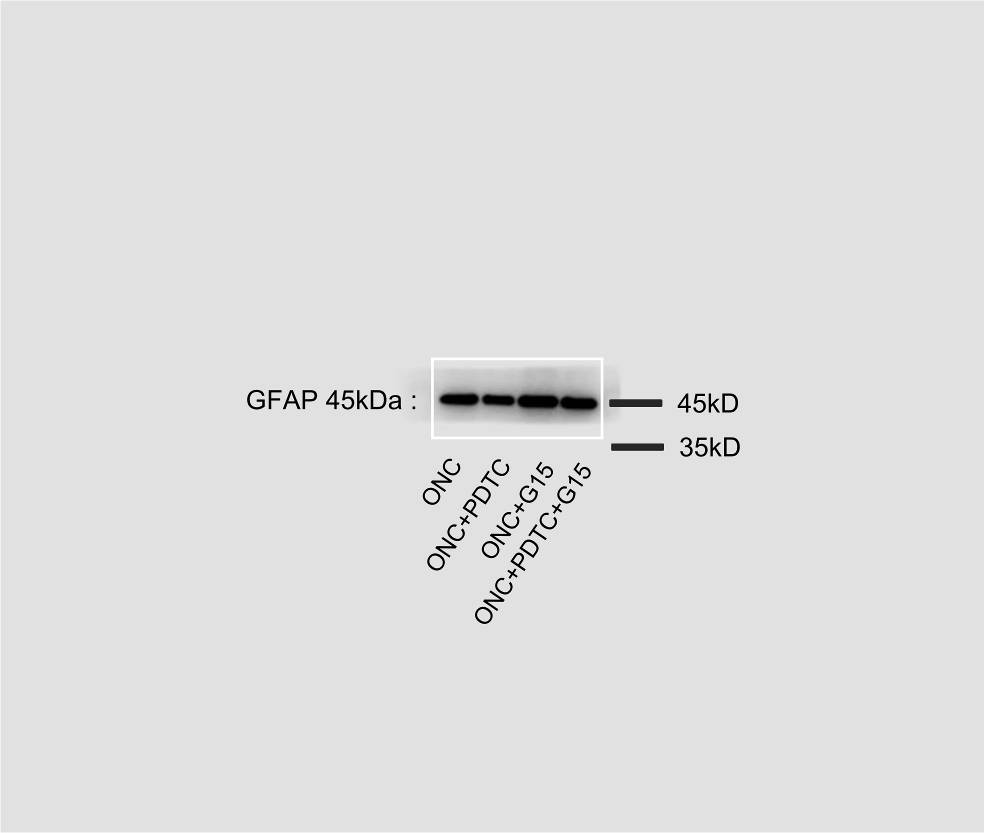
GFAP 45kDa :
45kD
35kD
ONC
ONC+PDTC
ONC+G15
ONC+PDTC+G15

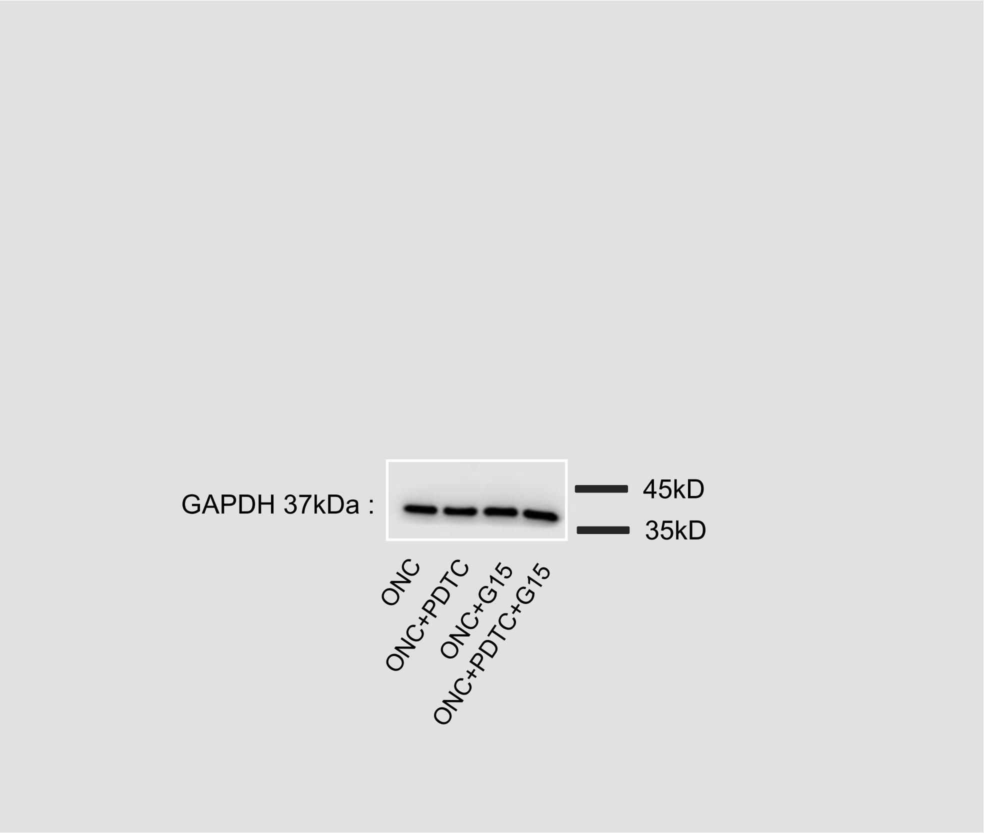
GAPDH 37kDa :
45kD
35kD
ONC
ONC+PDTC
ONC+G15
ONC+PDTC+G15

figures 7A

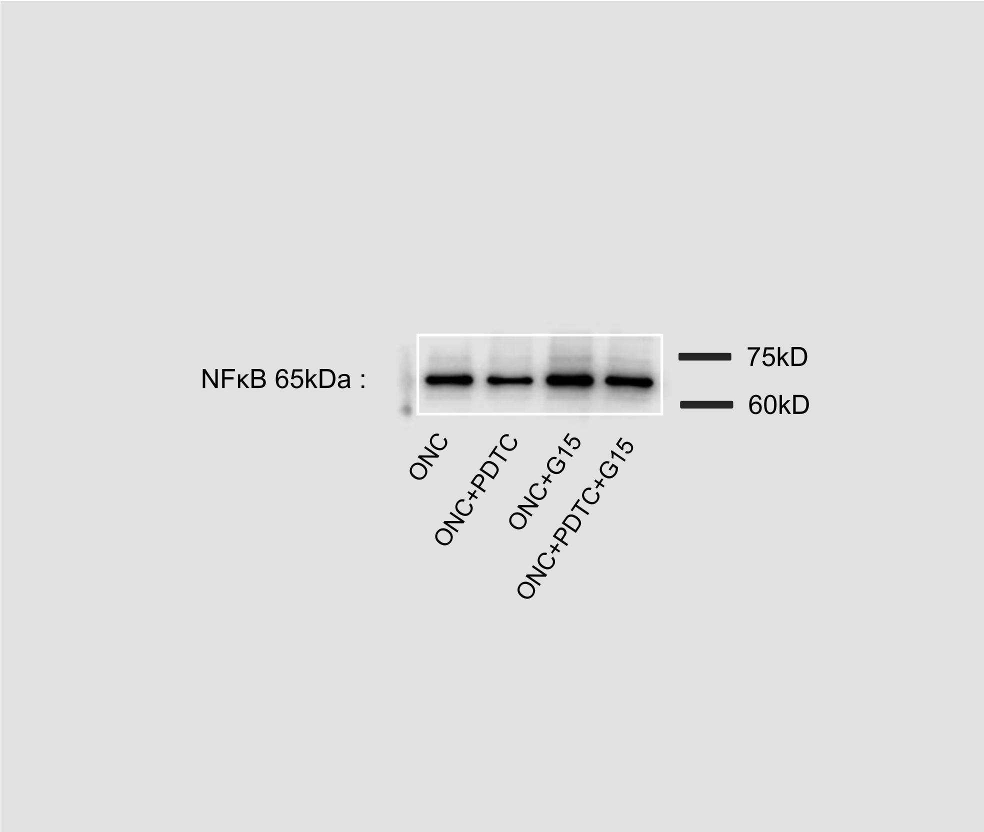
NFκB 65kDa :
75kD
60kD
ONC
ONC+PDTC
ONC+G15
ONC+PDTC+G15

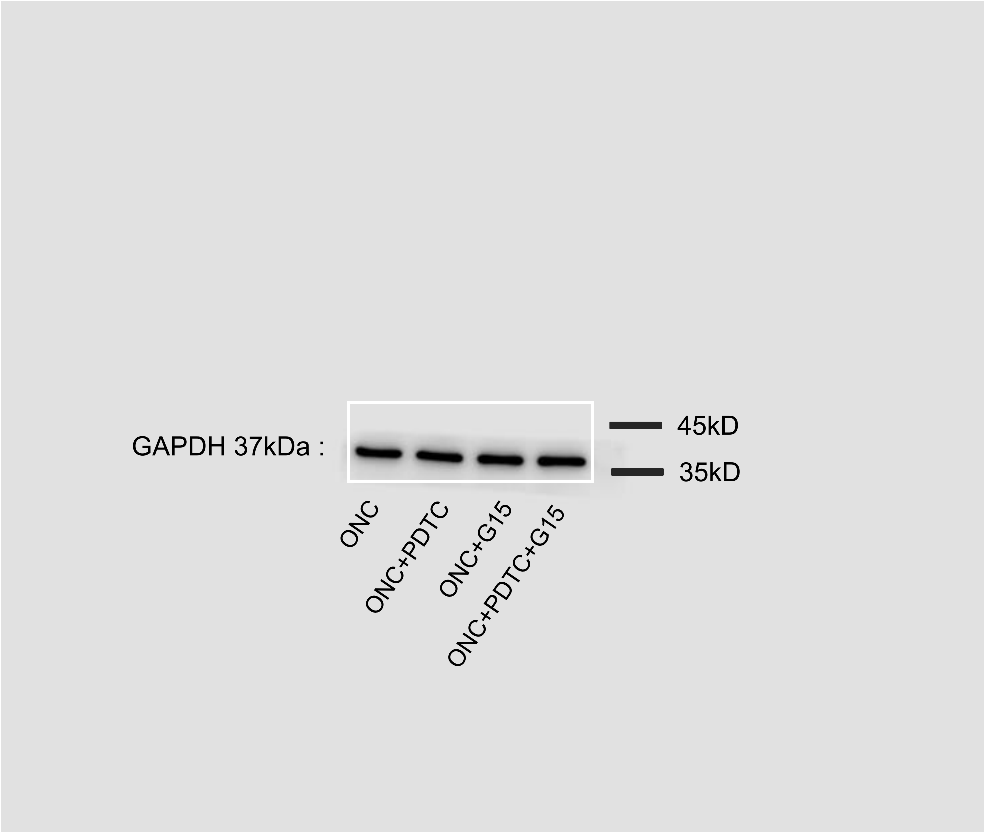
GAPDH 37kDa :
45kD
35kD
ONC
ONC+PDTC
ONC+G15
ONC+PDTC+G15

figures 7A

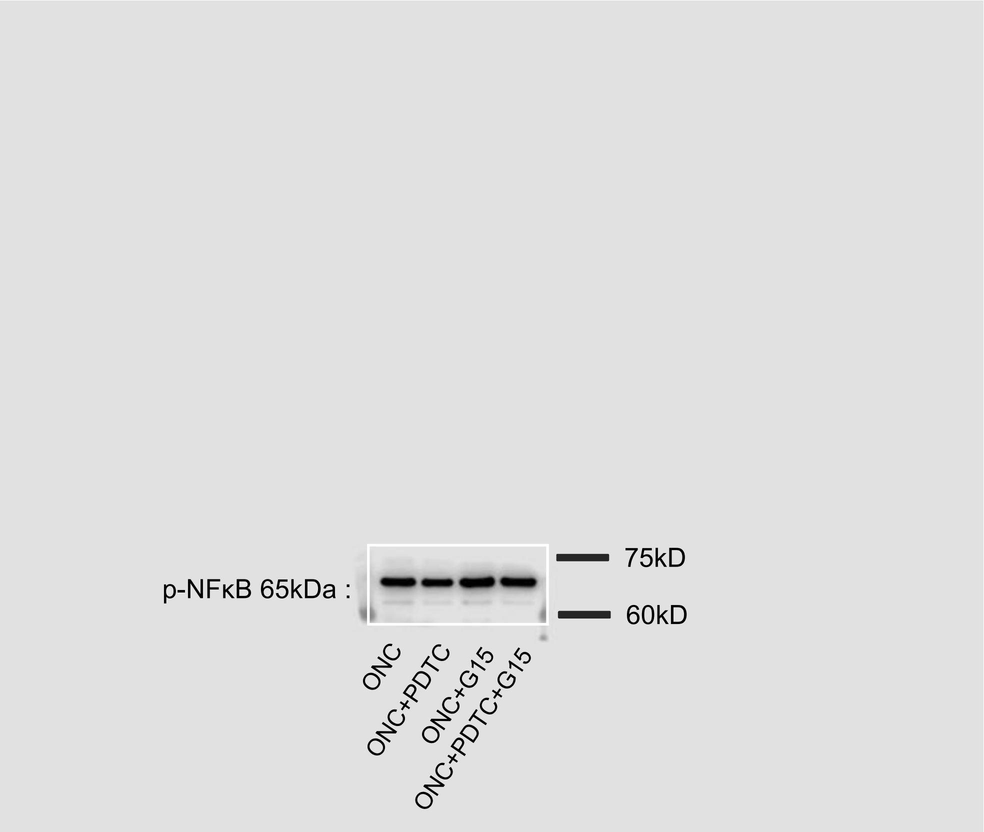
p-NFκB 65kDa :
75kD
60kD
ONC
ONC+PDTC
ONC+G15
ONC+PDTC+G15

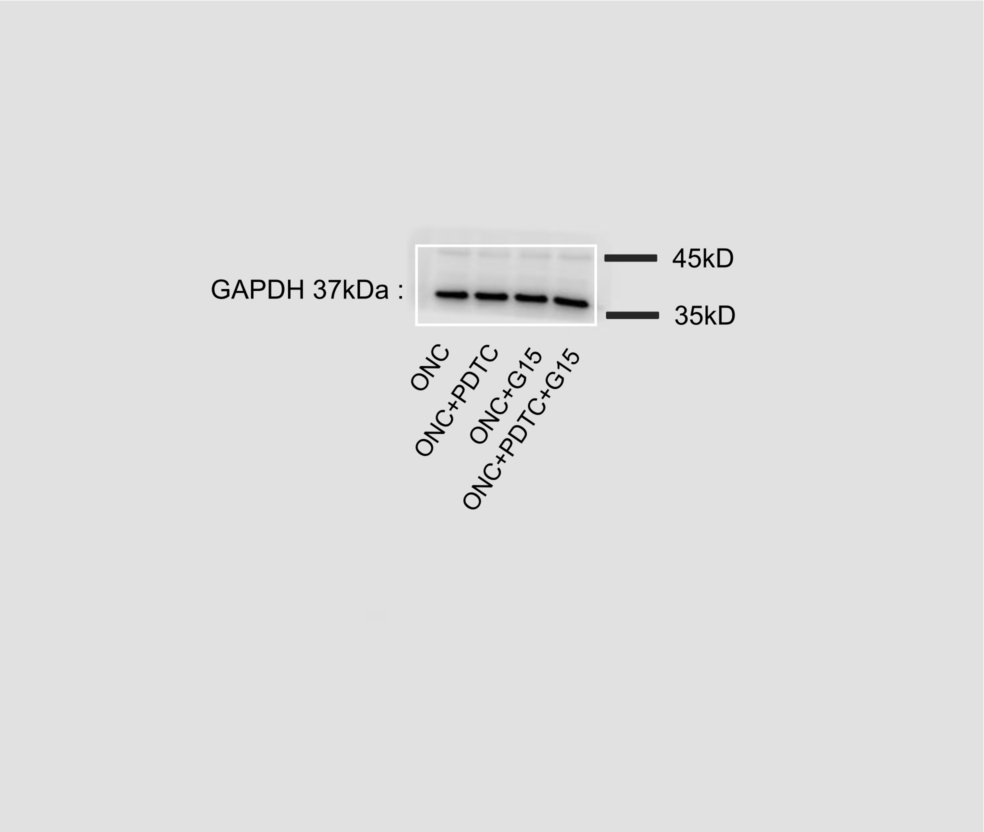
GAPDH 37kDa :
45kD
35kD
ONC
ONC+PDTC
ONC+G15
ONC+PDTC+G15

figures 7A

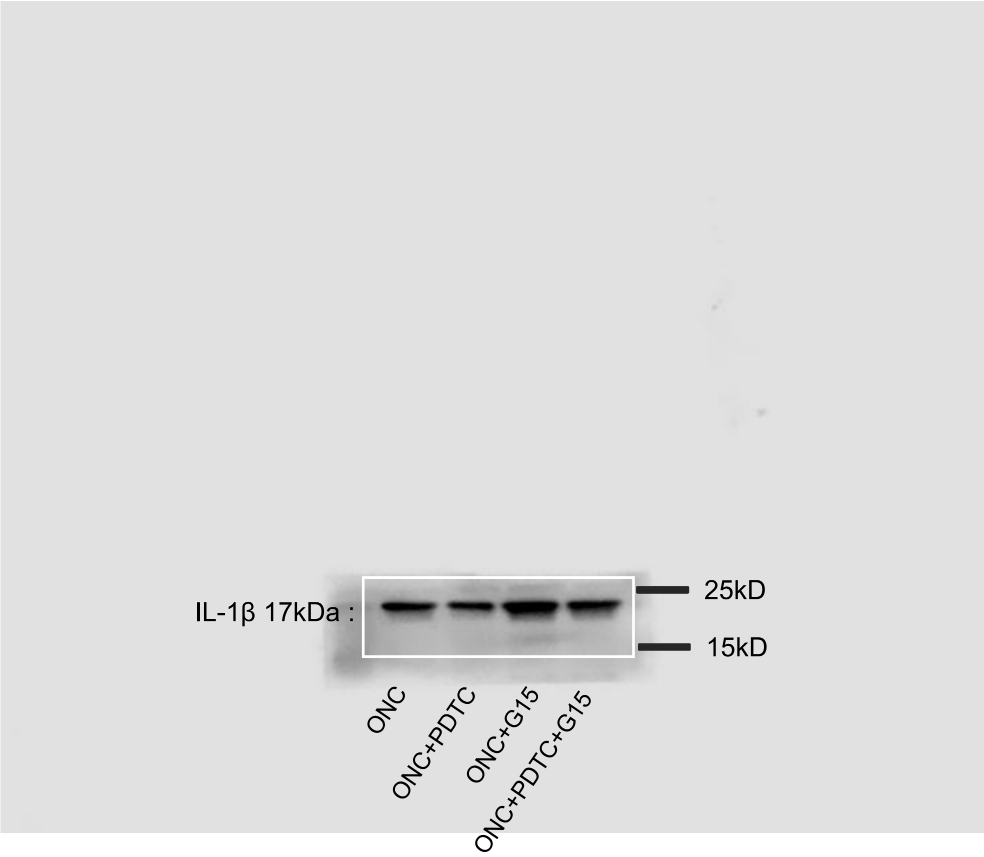
IL-1β 17kDa :
25kD
15kD
ONC
ONC+PDTC
ONC+G15
ONC+PDTC+G15

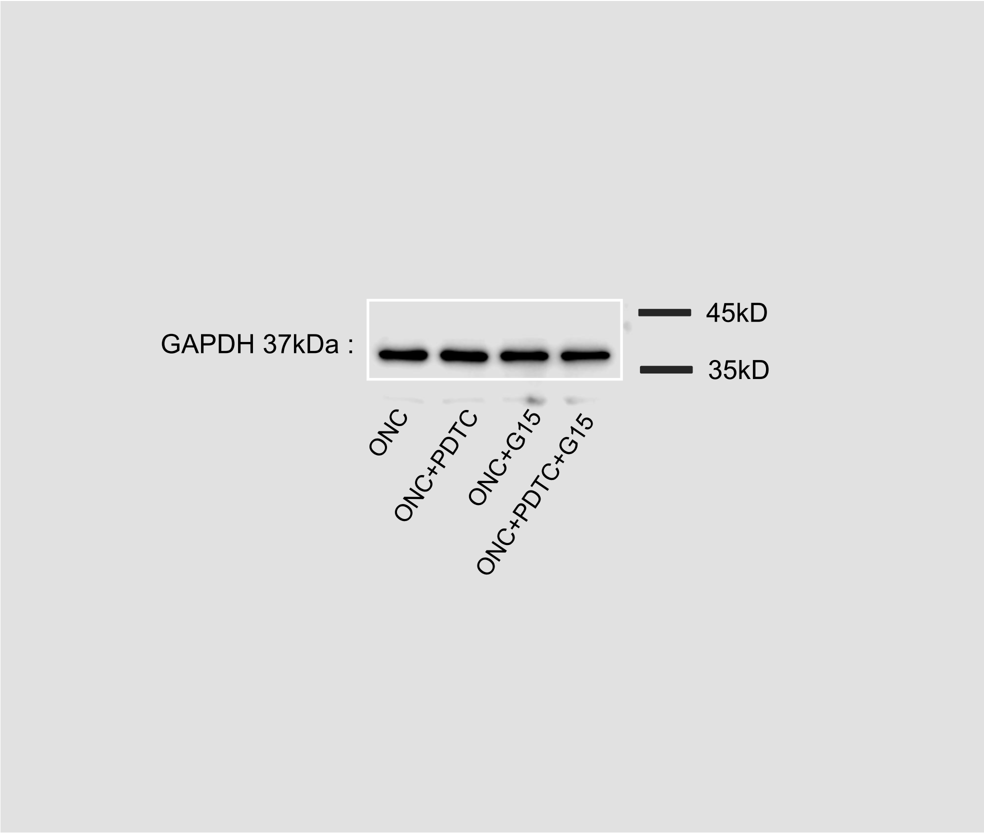
GAPDH 37kDa :
45kD
35kD
ONC
ONC+PDTC
ONC+G15
ONC+PDTC+G15

figures 7A

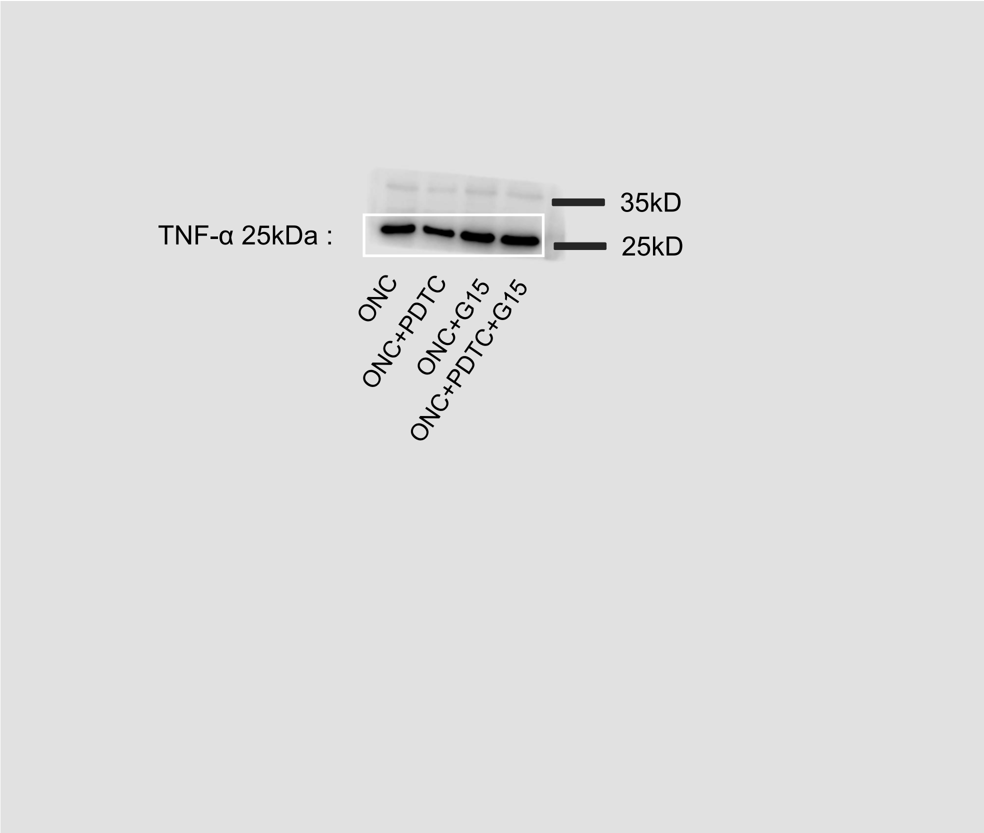

TNF-α 25kDa :
35kD
25kD
ONC
ONC+PDTC
ONC+G15
ONC+PDTC+G15

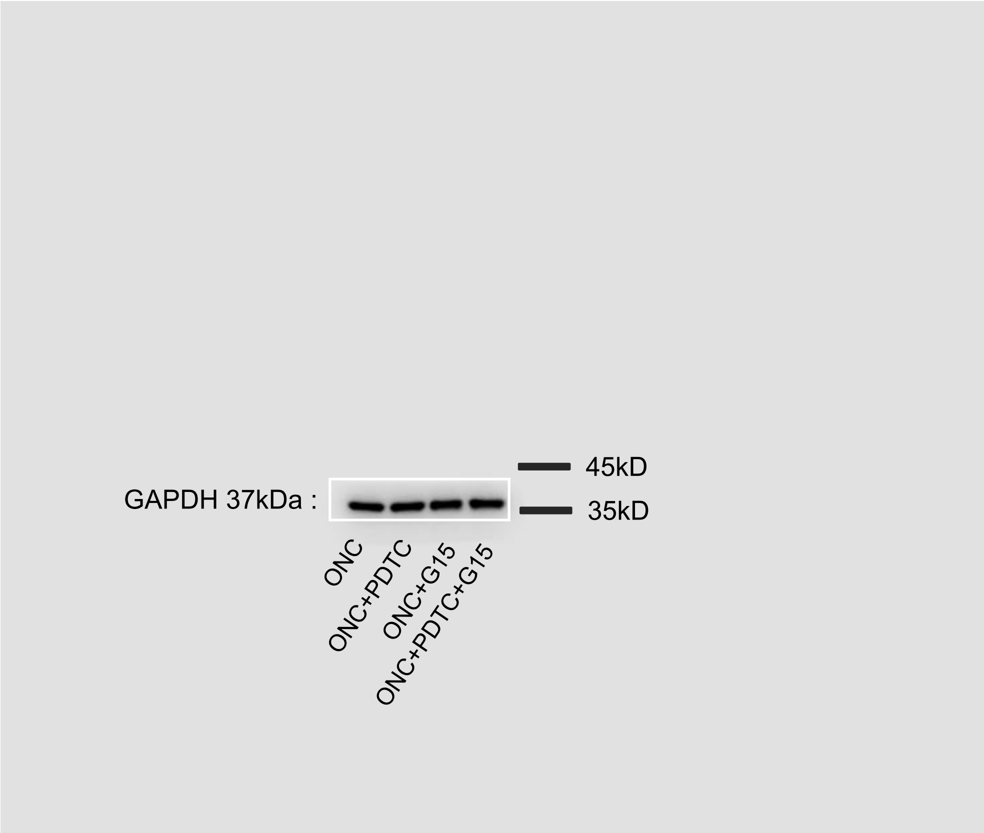

GAPDH 37kDa :
45kD
35kD
ONC
ONC+PDTC
ONC+G15
ONC+PDTC+G15

supplementary figures 2C

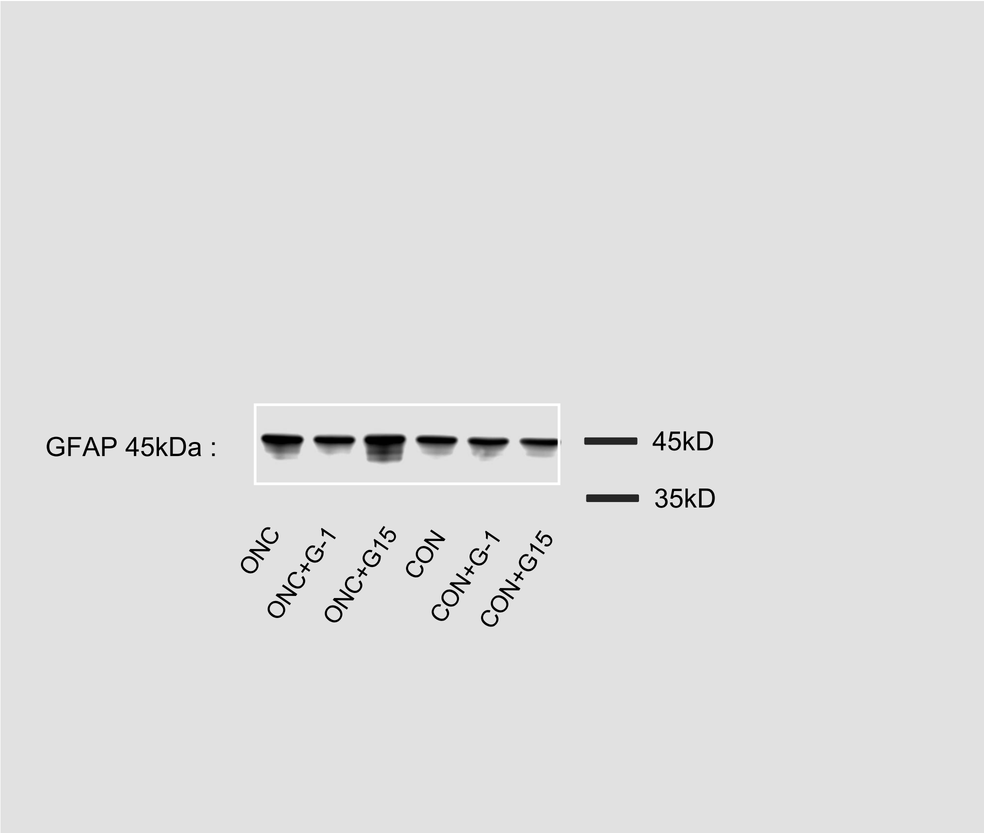

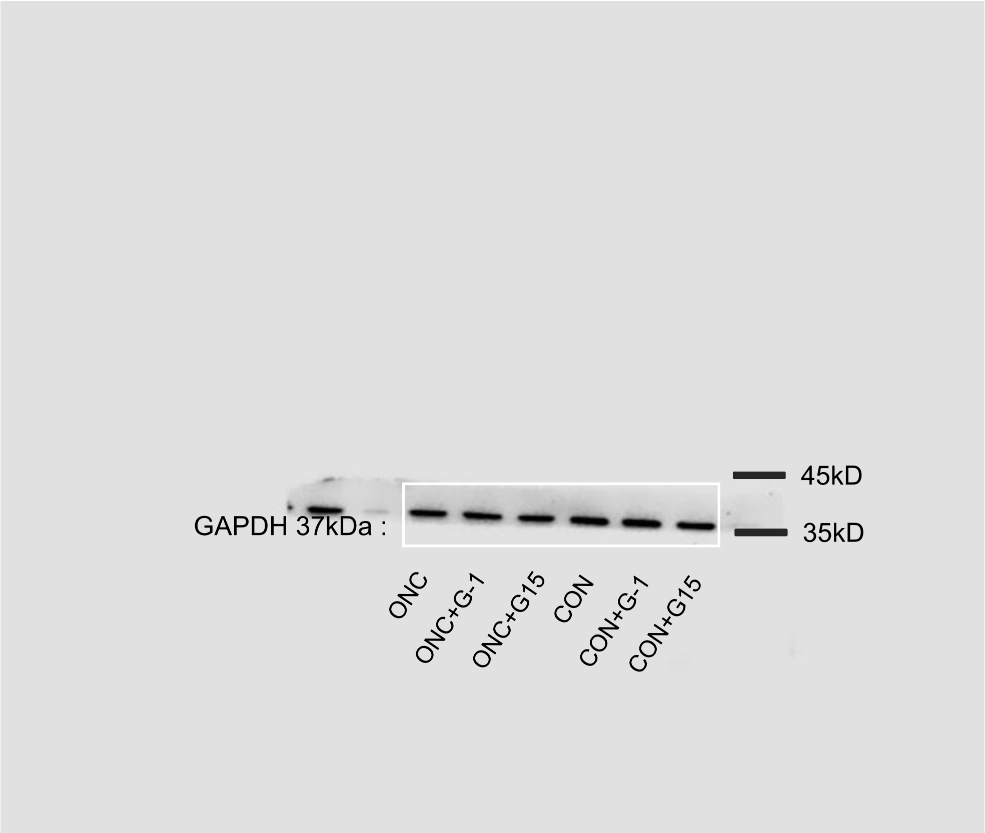

Supplement: Multimedia component 2 [file mmc2.pdf]
